# Radiosynthesis and biological evaluation of [^18^F]AG-120 for PET imaging of the mutant isocitrate dehydrogenase 1 in glioma

**DOI:** 10.1007/s00259-023-06515-7

**Published:** 2023-11-20

**Authors:** Thu Hang Lai, Barbara Wenzel, Sladjana Dukić-Stefanović, Rodrigo Teodoro, Lucie Arnaud, Aurélie Maisonial-Besset, Valérie Weber, Rareş-Petru Moldovan, Sebastian Meister, Jens Pietzsch, Klaus Kopka, Tareq A. Juratli, Winnie Deuther-Conrad, Magali Toussaint

**Affiliations:** 1 Institute of Radiopharmaceutical Cancer Research, Department of Neuroradiopharmaceuticals, Helmholtz-Zentrum Dresden-Rossendorf, Research site Leipzig, Leipzig, Germany; 2 Department of Research and Development, ROTOP Pharmaka GmbH, Dresden, Germany; 3 Université Clermont Auvergne, Imagerie Moléculaire et Stratégies Théranostiques, UMR 1240, Inserm, Clermont- Ferrand, France; 4 Institute of Radiopharmaceutical Cancer Research, Department of Radiopharmaceutical and Chemical Biology, Helmholtz-Zentrum Dresden-Rossendorf, Dresden, Germany; 5 School of Science, Faculty of Chemistry and Food Chemistry, Technische Universität Dresden, Dresden, Germany; 6 German Cancer Consortium (DKTK), Partner Site Dresden, Dresden, Germany; 7 National Center for Tumor Diseases (NCT) Dresden, University Hospital Carl Gustav Carus, Dresden, Germany; 8 Department of Neurosurgery, Faculty of Medicine, University Hospital Carl Gustav Carus, Technische Universität Dresden, Dresden, Germany

**Keywords:** IDH mutation, Ivosidenib, Malignant brain tumors, Orthotopic glioma xenograft model, Fluorine-18, CMRF, [^18^F]FET

## Abstract

Glioma are clinically challenging tumors due to their location and invasiveness nature, which often hinder complete surgical resection. The evaluation of the isocitrate dehydrogenase mutation status has become crucial for effective patient stratification. Through a transdisciplinary approach, we have developed an ^18^F-labeled ligand for non-invasive assessment of the IDH1R132H variant by using positron emission tomography (PET) imaging. In this study, we have successfully prepared diastereomerically pure [^18^F]**AG-120** by copper-mediated radiofluorination of the stannyl precursor **6** on a TRACERlab FX2 N radiosynthesis module. In vitro internalization studies demonstrated significantly higher uptake of [^18^F]**AG-120** in U251 human high-grade glioma cells with stable overexpression of mutant IDH1 (IDH1R132H) compared to their wild-type IDH1 counterpart (0.4 vs. 0.013% applied dose/µg protein at 120 min). In vivo studies conducted in mice, exhibited the excellent metabolic stability of [^18^F]**AG-120**, with parent fractions of 85% and 91% in plasma and brain at 30 min p.i., respectively. Dynamic PET studies with [^18^F]**AG-120** in naïve mice and orthotopic glioma rat model reveal limited blood-brain barrier permeation along with a low uptake in the brain tumor. Interestingly, there was no significant difference in uptake between mutant IDH1R132H and wild-type IDH1 tumors (tumor-to-blood ratio_[40−60 min]_: ~1.7 vs. ~1.3). In conclusion, our preclinical evaluation demonstrated a target-specific internalization of [^18^F]**AG-120** in vitro, a high metabolic stability in vivo in mice, and a slightly higher accumulation of activity in IDH1R132H-glioma compared to IDH1-glioma. Overall, our findings contribute to advancing the field of molecular imaging and encourage the evaluation of [^18^F]**AG-120** to improve diagnosis and management of glioma and other IDH1R132H-related tumors.

**Supplementary Information:**

The online version contains supplementary material available at 10.1007/s00259-023-06515-7.

## Introduction

Glioma represent a significant challenge in clinical practice as they account for approximately 80% of primary malignant brain tumors and currently lack a curative treatment [1]. Despite aggressive standard therapies with surgery, radiotherapy, and chemotherapy, as well as the development of novel therapies such as targeted therapies, electric field therapies and immunotherapies, the 5-year overall survival rate has not improved significantly in recent decades [2, 3]. Surgical approach prioritizes the preservation of neurological function over the extent of resection, often leading to systematic tumor recurrence [4, 5].

Mutations in the metabolic enzymes isocitrate dehydrogenase 1 and 2 (IDH1 and IDH2) occur in more than 70% of low-grade glioma and approximately 12% of high-grade glioma [6]. These somatic gene mutations involve a heterozygous missense substitution of the purine base guanine at position 395, typically transitioning through the purine base adenine (G395A). The most common subtype, found in 90% of the cases, is an arginine-to-histidine substitution (R132H) in the IDH1 isoform (IDH1R132H) [6–8]. These gain-of-function mutations induce a neomorphic activity by the NADPH-dependent conversion of *alpha*-ketoglutarate (*α*-KG) to D-2-hydroxyglutarate (2-HG). Intracellular accumulation of the 2-HG, > 100-fold higher than in normal tissue [9, 10], and intracellular depletion of NADPH lead to a redox imbalance and metabolic and epigenetic reprogramming, which are thought to contribute to gliomagenesis [11]. Indeed, the IDH mutation (mIDH) has been identified as an early event in gliomagenesis preceding secondary and tertiary genetic alterations [11, 12], retained during progression [13–15] and remarkably ubiquitously expressed by tumor cells, including infiltrating single cells [16–19]. Therefore, since 2016, the detection of the mIDH has redefined the landscape of glioma management [17], offering a more precise patient stratification based on its prognostic and predictive value [18, 19].

The development of mIDH inhibitors, particularly of the IDH1 subtype, has rapidly expanded [20]. Almost all the small-molecule inhibitors developed to date interfere with the enzymatic reaction of the mutant enzyme through allosteric binding. More than nine potential drugs are currently in clinical trials for various cancers, and the mIDH1 inhibitor **AG-120** (ivosidenib) received FDA approval in 2019 for treatment of newly diagnosed acute myeloid leukemia and advanced cholangiocarcinoma. In addition, the INDIGO clinical trial (NCT04164901) demonstrated the potential of **AG-881** (vorasidenib) for treating low-grade glioma [20].

Apart from therapeutic applications, mIDH also offers an attractive target for noninvasive tumor characterization through nuclear imaging techniques in glioma patients [21–24]. Currently, the direct immunohistochemical detection of mIDH is recommended, followed by next-generation sequencing if the result is negative [4]. An alternative indirect method is the non-invasive measurement of 2-HG by magnetic resonance spectroscopy; however, this technique suffers from limited spatial resolution and availability [25].

Positron emission tomography (PET) imaging of dopamine metabolism using [^18^F]**FDOPA** or of L-amino acid uptake using [^18^F]**FET**, has been investigated for the diagnosis, prognosis and assessment of treatment-related changes in mIDH glioma [26–29]. However, their applicability for mIDH detection is limited due to their indirect correlation to the IDH status and to a large fraction of [^18^F]**FET**-PET-negative low-grade glioma [30–34]. The non-invasive direct imaging of mutant IDH1R132H tumors could not only provide an alternative diagnostic tool for the 20% of patients whose IDH status cannot be specified otherwise [35, 36], but also support the development of mIDH-targeted therapies by assessing target engagement and treatment response. Furthermore, the unique feature of mIDH being present throughout the tumor and absent in normal tissue allows for improved identification of tumor recurrence *versus* treatment-related changes. Recently reviews by Neumaier et al. have highlighted the development of highly potent and selective pharmacological mIDH inhibitors encouraging the development of radiotracer imaging agents [24]. However, in vivo evaluations have been reported for only a few of the ^14^C-, ^18^F- and ^125^I-labeled mIDH inhibitors developed to date [37–40], without successful neuroimaging to the best of our knowledge. Also the recent study by Wang et al. reporting the development of [^18^F]**AG-120** as a mixture of the *S,S*-diastereomer (**AG-120**) and the *S,R*-diastereomer together with the mutant-specific accumulation of the radiotracer in peripheral mIDH-positive tumors, did not focus specifically on brain imaging [40].

In the present study, we successfully prepared the stereoisomerically pure radioligand [^18^F]**AG-120** and assessed its performance in vitro through binding affinity and internalization studies. Additionally, pharmacokinetic and metabolism studies were conducted in naïve mice and dynamic PET imaging was performed in a preclinical rat model of orthotopic glioma overexpressing the mutant IDH1R132H or the wild-type IDH1.

## Materials and methods

See Supplementary Information (SI) for full description of all materials and methods, including organic and radiochemistry procedures, next-generation sequencing, potency assays and immunoassays.

### Radiosynthesis

Automated productions of [^18^F]**AG-120** and [^18^F]**FET** were performed on a TRACERlab FX2 N radiosynthesizer (GE Healthcare, USA). For the copper-mediated radiofluorination (CMRF) of the stannyl precursor **6**, different reaction parameters such as fluorination agent, solvent or temperature were systematically investigated and optimized.

### Cell culture

Stably transfected U251-MG cells (originating from a human glioblastoma) overexpressing human wild-type IDH1 (IDH1-U251) or human mutant IDH1R132H (IDH1R132H-U251), (Fig. S5) were generated by Dr. Jacqueline Kessler and Prof. Dirk Vordermark (Klinik für Strahlentherapie am Universitätsklinikum Halle Saale, Germany) [41].

### In vitro binding assays

All binding experiments were performed by incubation of [^18^F]**AG-120** with lysates obtained from transfected U251 cells and were terminated by filtration through two layers of GF55 glass fiber filters (Hahnemühle FineArt GmbH, Dassel, Germany). Data analysis was performed by nonlinear regression using GraphPad Prism software.

### In vitro cell uptake

Transfected U251 cells were seeded in 24-well plates one day before the experiment and inhibitors were added 2 h prior to radiotracer. The uptake study was performed at 37 °C in a CO_2_-incubator. Cell surface-bound activity was released by treatment with acid-glycine buffer (0.2 M glycine, 0.15 M NaCl, pH 3) prior to cell lysis (0.1 M NaOH, 1% SDS). The concentrations of surface-bound and internalized activity per well were calculated as a percentage of the applied dose (AD) per well and normalized to the protein concentration per well (% AD/µg protein).

### In vivo metabolism

All animal experiments are in compliance with the EU Directive 2010/63 and were approved by the local authority. [^18^F]**AG-120** (27 ± 7 MBq; 13 ± 5 nmol/kg) was administered intravenously in awake female CD-1 mice (n = 3), and plasma and brain samples were collected 30 min p.i. for reversed phase radio-HPLC (RP-HPLC) and micellar HPLC (MLC) analysis.

### Dynamic PET in naïve mice

Female CD-1 mice underwent a 60-min PET scan using a preclinical PET/MRI (NanoScan^®^, Mediso). [^18^F]**AG-120** (5.4 ± 0.7 MBq; 3.4 ± 2.3 nmol/kg) was administered by intravenous (i.v) injection 30 min after i.v. application of cyclosporine A (Sandimmune^®^, 50 mg/kg; n = 5), an inhibitor of the P-glycoprotein (P-gp) efflux transporter, or vehicle (NaCl/3% EtOH/6.6% kolliphor; n = 4). Data were analysed using PMOD v3.9 and are expressed as mean standardized uptake value (SUV_mean_) of the respective entire region of interest (ROI).

### Dynamic PET in rats bearing glioma xenografts

Four nude rats were orthotopically injected with 1 × 10^6^ IDH1- or 5 × 10^6^ IDH1R132H-U251 cells (n = 2 per group) and imaging studies were performed on day 30 and 34 post-graft. [^18^F]**AG-120** (38.7 ± 1.5 MBq; 1.5 ± 0.1 nmol/kg) or [^18^F]**FET** (36.6 ± 1.0 MBq; 12.0 ± 0.3 nmol/kg) was administered by tail vein injection followed by a 60-min PET/CT scan (NanoScan^®^, Mediso), and data were analyzed using PMOD v3.9. The tumor region was delineated from the T2-weighted image (7T MRI, BioSpec 70/30, Bruker). Background signal was defined as a 1.5 mm sphere placed in the contralateral (left striatum), and region-specific time-activity curves (TACs) were generated. Tumor-to-background ratios were calculated for the 30–60 min time frame (TBR_mean_=SUV_mean_(tumor)/SUV_mean_(contralateral)).

## Results

### Radiosynthesis of [^18^F]AG-120

**Scheme 1:**
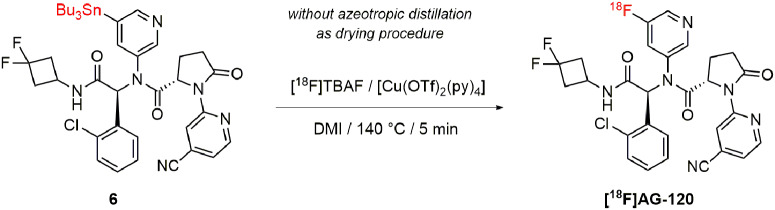
Copper-mediated radiofluorination of [^18^F]**AG-120**.

To optimize the reaction conditions for the copper-mediated radiofluorination of the stannyl precursor **6** (Scheme 1), different reaction parameters were tested in manual experiments according to published procedures [42–50]: (i) bases/salts combinations for elution of [^18^F]fluoride from the anion-exchange cartridge (TBAHCO_3_, NaOTf/K_2_CO_3_ [43], TBAOTf [44], DMAPHOTf [47–49]), (ii) respective fluorination agents ([^18^F]TBAF, [^18^F]DMAPHF, [^18^F]NaF), (iii) solvents (*N,N*-dimethylacetamide (DMA), 1,3-dimethyl-2-imidazolidinon (DMI)), (iv) temperatures (115–140 °C) and (v) the need for conventional drying of the [^18^F]fluoride by azeotropic distillation with acetonitrile (details are in the SI and in Tab. S1). Due to the complex synthesis of the stannyl precursor **6**, only 3.0 mg (3.5 µmol) was used for the experiments. The copper complex [Cu(OTf)_2_(py)_4_] was chosen as mediator for the radiofluorination of **6**. In summary, the highest radiochemical conversion (RCC) of 10% was achieved using DMI as solvent and [^18^F]TBAF, which was obtained by the elution of the [^18^F]fluoride from a Chromafix 30 PS-HCO3 cartridge with TBAOTf dissolved in anhydrous methanol [42, 44]. Evaporation of the methanol within a few minutes eliminated the need for azeotropic distillation. At 140 °C, the radiolabeling reaction was completed after only 5 min (Scheme 1). These labeling conditions were used for the development of an automated radiosynthesis of [^18^F]**AG-120** using the TRACERlab FX2 N radiosynthesis module (details are provided in SI and Fig. S2). In this automated procedure, purification of the crude radiolabeling mixture was performed by solid phase extraction (SPE) on a C18 light cartridge to eliminate excess of copper impurities, followed by semi-preparative HPLC (Fig. S3). To remove the HPLC solvent, another SPE was performed and the radiotracer was formulated in isotonic saline containing 10% ethanol (~ 1 MBq/µL). In a total synthesis time of ~ 75 min, [^18^F]**AG-120** was produced with a high radiochemical purity of ≥ 99%, a radiochemical yield of 3.8 ± 0.3% (n = 9, EOB) and molar activities in the range of 80–160 GBq/µmol (n = 6, EOS) with starting activities of 10–30 GBq. The identity and stereoisomeric purity of [^18^F]**AG-120** was confirmed by analytical radio- and UV-HPLC by co-injection of the final radiotracer with (i) commercially available **AG-120** (*S,S*-diastereomer) and (ii) a mixture of the *S,S-* and *S,R*-diastereomers (Fig. S4). Interestingly, the separation of the two diastereomers was only observed using the isocratic mode.

### Kinetics, affinity and specificity of the binding of [^18^F]AG-120

To establish a protocol for the first measurement of the binding affinity of the allosteric mIDH inhibitor **AG-120** for IDH1R132H, we initially investigated the effects of the components of the buffer used in the potency studies for the binding measurements (Fig. S6). For all tested buffer compositions (Tab. S2), the total binding in IDH1R132H-U251 cell lysates was comparable and remarkably exceeded that obtained with IDH1-U251 cells lysates (Fig. S7), and we performed the subsequent experiments with PBS supplemented with 10 mM MgCl_2_ because divalent cations have to be present in the active site for catalysis [51–53].

**Fig. 1:**
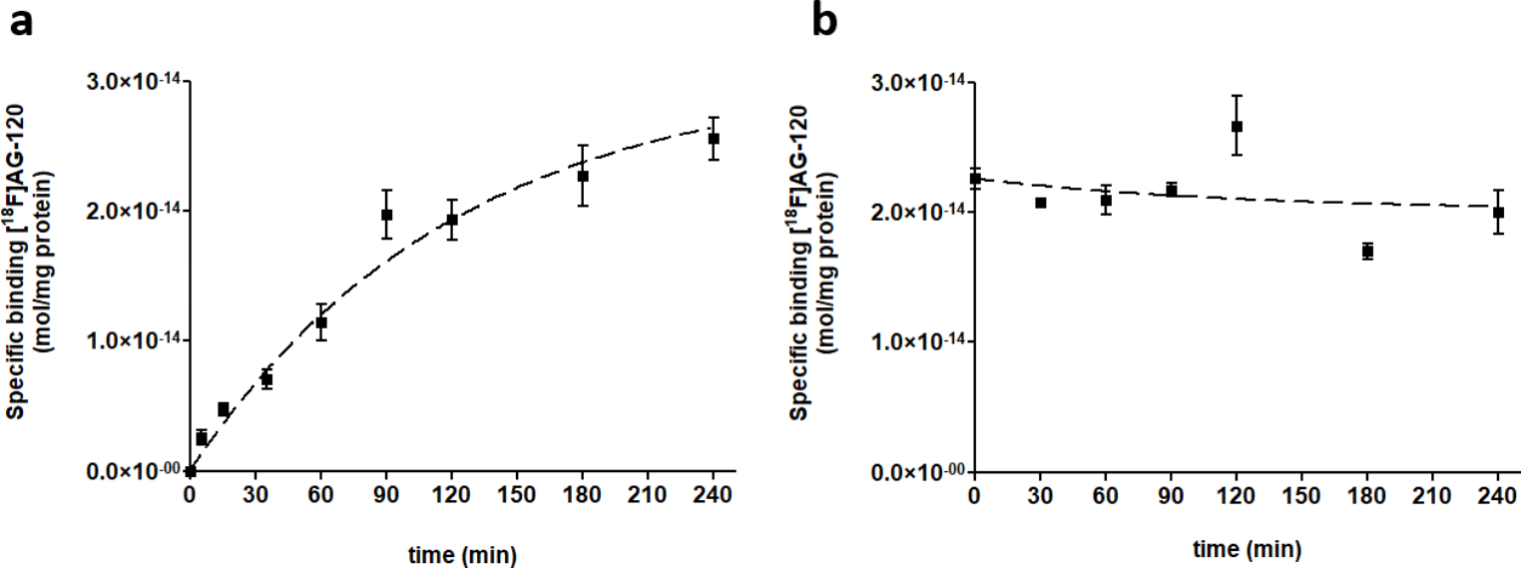
Association (**a**) and dissociation (**b**) of [^18^F]**AG-120** with IDH1R132H-U251 cell lysate. After incubation of [^18^F]**AG-120** for 60 min, the dissociation was initiated by the addition of **BAY1436032** (1 µM). Non-specific binding was determined by co-incubation with **BAY1436032** (1 µM). Data were fitted best by the one-phase exponential association equation (r^2^ = 0.9160)

To determine the incubation time required to measure the specific binding of [^18^F]**AG-120** at equilibrium, we investigated the kinetics of the association and dissociation of [^18^F]**AG-120** with IDH1R132H-U251 whole cell lysates. The studies reproducibly showed that the specific binding of [^18^F]**AG-120** at low nanomolar concentrations reached a plateau with a half-life of approximately 60 min (Fig. 1a). However, this binding appears to be irreversible. Neither the addition of **AG-120** to minimize the effect of potential rebinding nor the addition of **BAY1436032**, as a structurally different allosteric IDH1R132H inhibitor in excess after a 60-min association period, resulted in any relevant dissociation of specifically bound radioligand during the 4-h study (Fig. 1b).

**Fig. 2:**
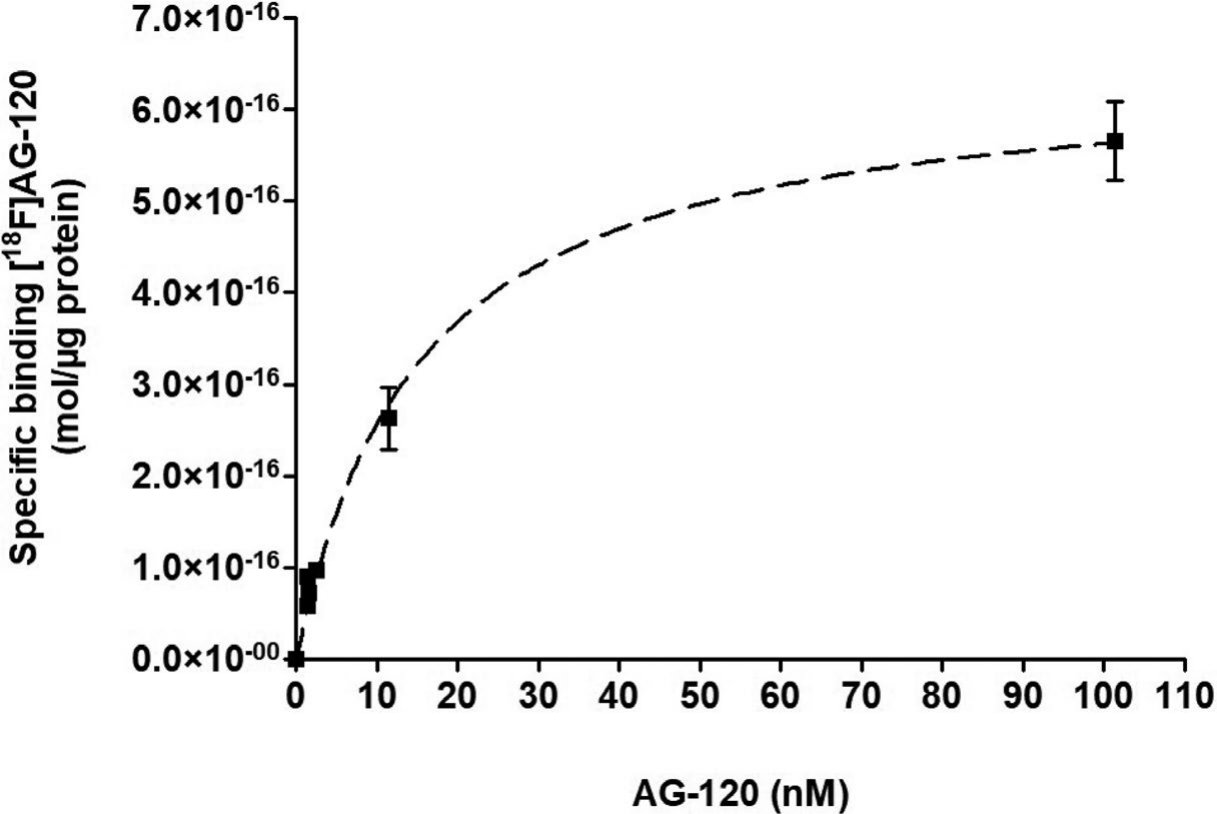
Saturation binding curve for [^18^F]**AG-120** with IDH1R132H-U251 cell lysate. Lysates were incubated with 1.44 nM [^18^F]**AG-120** for 60 min. Non-specific binding was determined by co-incubation with **BAY1436032** (1 µM). Data were fitted best by the one-site binding equation (r^2^ = 0.9644). *K*_D,app_=15.2 nM; apparent *B*_max,app_=648 fmol/mg protein

Taken together, the results indicate a comparatively slow formation of the [^18^F]**AG-120**:IDH1R132H complex and a long lifetime of this complex. Since it was not possible to determine the equilibrium dissociation constant *K*_D_ as the ratio of the dissociation rate constant *k*_off_ over the association rate constant *k*_on_, we performed homologous competition experiments with lysates of IDH1R132H-U251 cells to estimate the apparent *K*_D_ value. Nonlinear regression analysis of the corresponding saturation curve indicates specific binding of [^18^F]**AG-120** to a single target population with an apparent *K*_D_ value of 15 nM and an apparent *B*_max_ value of about 650 fmol/mg protein (Fig. 2). Accordingly, an apparent binding potential (BP = *B*_max_/*K*_D_) of about 4 can be calculated for the binding of [^18^F]**AG-120** in IDH1R132H-U251 cells.

To further evaluate the in vitro specificity of [^18^F]**AG-120**, a comparative homologous competition experiment was performed with IDH1- and IDH1R132H-U251 cell lysates (Fig. S8). The interaction strength of the radioligand appears to be in the same range for both isoforms, in contrast to the confirmed selective inhibition of the mutant enzyme by **AG-120** (Fig. S6). However, the apparent concentration of the binding sites in the IDH1R132H-U251 cells is approximately 5-fold higher than in the IDH1-U251 cells. Since the Western blot results (Fig. S5) indicate comparable expression densities of wild-type and mutant IDH1 in the respective stably transfected cells, the estimated higher binding potential of [^18^F]**AG-120** in the IDH1R132H- compared to the IDH1-U251 cells could be translated into a meaningful signal-to-background ratio in vivo.

### Kinetics and specificity of the cellular uptake of [^18^F]AG-120

**Fig. 3:**
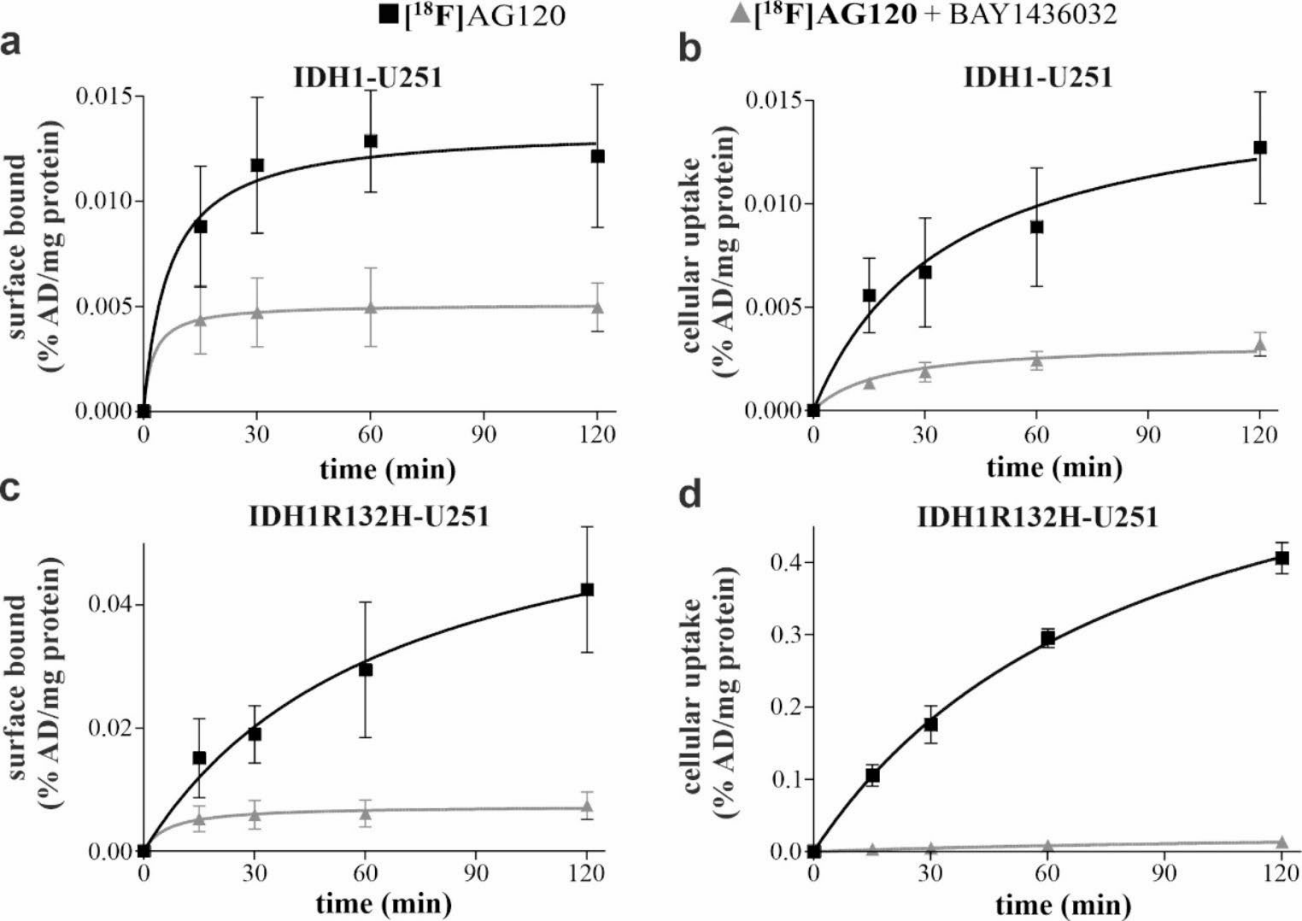
Cellular uptake of [^18^F]**AG-120** in IDH1-U251 (**a, b**) and IDH1R132H-U251 (**c, d**) cells. Surface-bound (**a, c**) and internalized radioligand fractions (**b, d**) kinetics in IDH1-U251 or IDH1R132-U251 pre-incubated (2 h) with vehicle (0.01% DMSO; control) or **BAY1436032** (1 µM) before the addition of [^18^F]**AG-120** (6.1 ± 1.1 nM). Results are presented as % of applied dose of the radioligand per mg protein (% AD/mg protein) vs. incubation time. All curves fitted best with a one-site model. Data were obtained from 4–5 independent experiments

To ascertain whether the results obtained with whole cell lysates were reproducible in a test system of higher complexity, uptake, binding and release studies were performed using living IDH1- or IDH1R132H-U251 cells. The data demonstrated a much higher [^18^F]**AG-120** cellular uptake in IDH1R132H-U251 compared to IDH1-U251 cells (Fig. 3).

The association of [^18^F]**AG-120** with the cell surface was faster in IDH1-U251 cells compared to IDH1R132H-U251 cells with an association half-life of 8 vs. 38 min. In both cell lines, internalization was slower than association, corresponding to an internalization of previously cell surface-bound activity. However, while the proportions of surface bound and internalized activity in IDH1-U251 cells appear to be equivalent (approximately 0.013% AD/mg protein at 120 min incubation), the intracellular level of activity in IDH1R132H-U251 cells was about ten times higher than the concentration of surface-bound activity (0.4% vs. 0.04% AD/mg protein at 120 min). Pre-administration of the pan-mIDH1 inhibitor **BAY1436032** in excess not only considerably reduced [^18^F]**AG-120** binding to the surface of both IDH1- and IDH1R132H-U251 cells to similar levels, but also shortened the time to plateau. Overall, the stably transfected cells appear to have a specific binding site for mIDH inhibitors on their membrane that is involved in the internalization of the inhibitor. Furthermore, the relatively slow but constantly increasing accumulation of activity in IDH1R132H-U251, at levels well above the equilibrium between associated and internalized activity, indicates a high intracellular concentration of [^18^F]**AG-120** binding sites, which is not saturable within the 2-h incubation period.

**Fig. 4:**
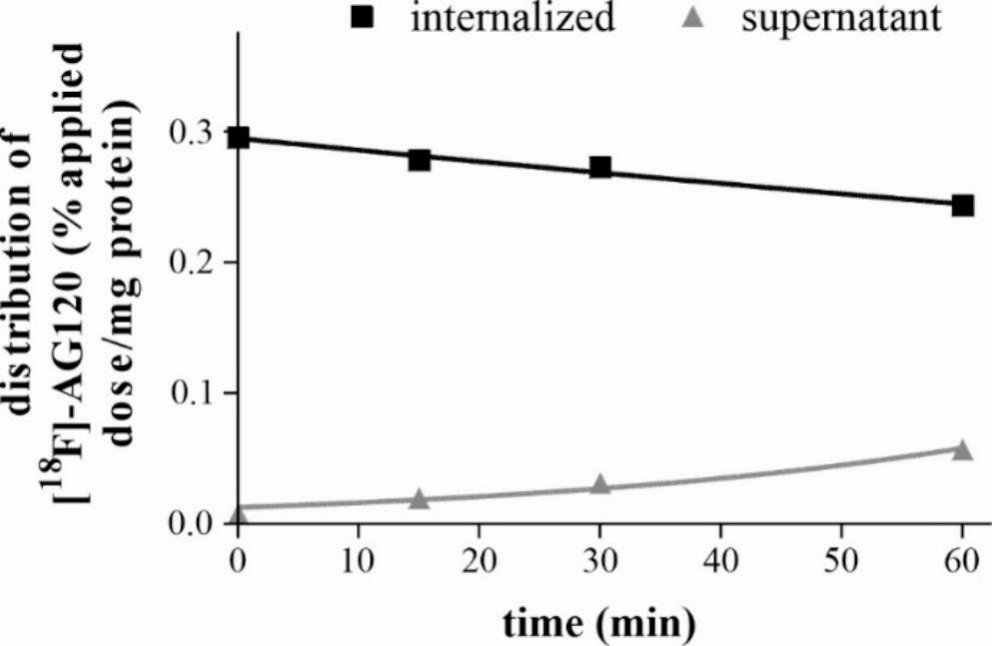
Efflux of [^18^F]**AG-120** from IDH1R132H-U251 cells after 60 min pre-incubation (1.23 nM [^18^F]**AG-120**) at 37 °C. Results are presented as % of applied dose of the radioligand per mg protein (% AD/mg protein) vs. incubation time

To further evaluate the suitability of [^18^F]**AG-120** for in vivo biodistribution studies, we investigated whether the internalized radioligand is released from the cells (Fig. 4). Less than 10% of the initially taken up activity is released during the 1-h incubation period, corresponding to the absence of any relevant dissociation observed in the kinetic experiments with lysates indicating a high intracellular retention of [^18^F]**AG-120**.

### [^18^F]AG-120 metabolism in naïve mice

**Fig. 5:**
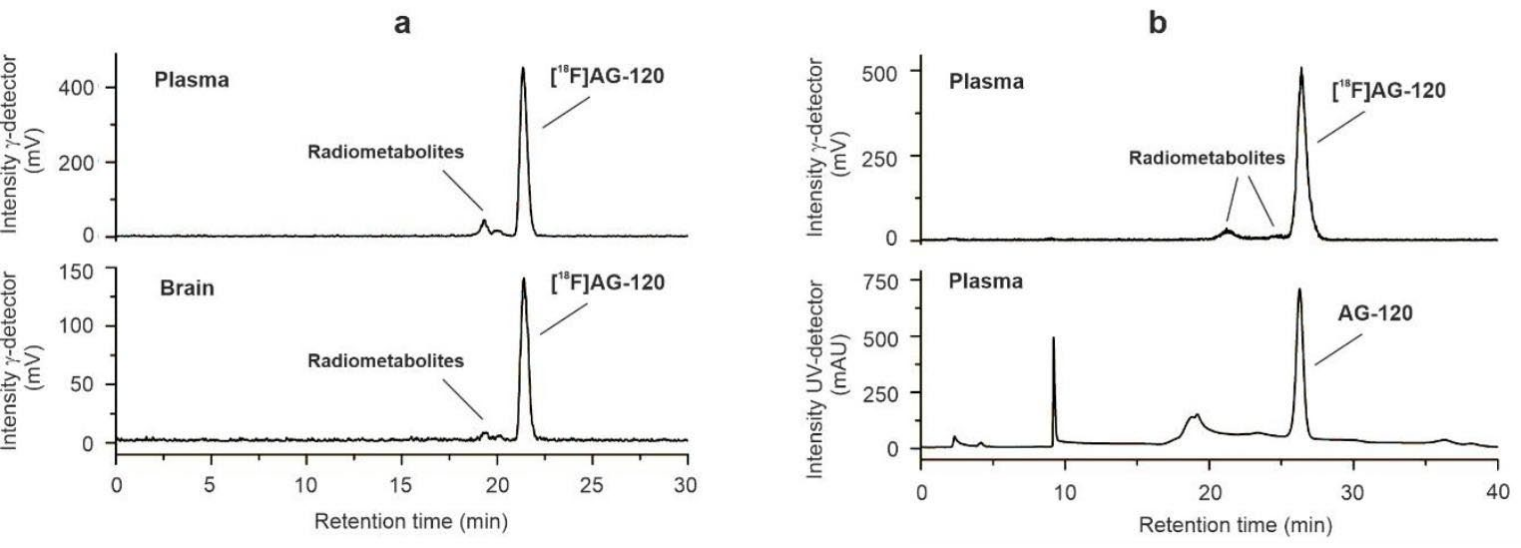
Radiometabolites analyses of [^18^]**AG-120** in mouse plasma and brain at 30 min p.i. (**a**) Radio-chromatograms of extracts of mouse plasma and brain samples measured by analytical RP-HPLC. (**b**) Radio- and UV-chromatogram of a mouse plasma sample spiked with reference **AG-120** measured by analytical MLC.

We investigated the metabolic stability of [^18^F]**AG-120** by radio-RP-HPLC and radio-MLC analyses of plasma and brain samples obtained from mice (n = 3) at 30 min post i.v. injection of [^18^F]**AG-120**. Activity recoveries were always > 90%, reflecting the efficiency of the extraction protocols. The parent fraction was 86 ± 2% and 91 ± 1% in the plasma and brain, respectively, indicating a high metabolic stability of [^18^F]**AG-120** in vivo (Fig. 5). Although we found two radiometabolites in the brain samples, a relevant contribution to the PET signal in the brain is unlikely due to their extremely low concentration.

### Biodistribution in naïve mice

**Fig. 6:**
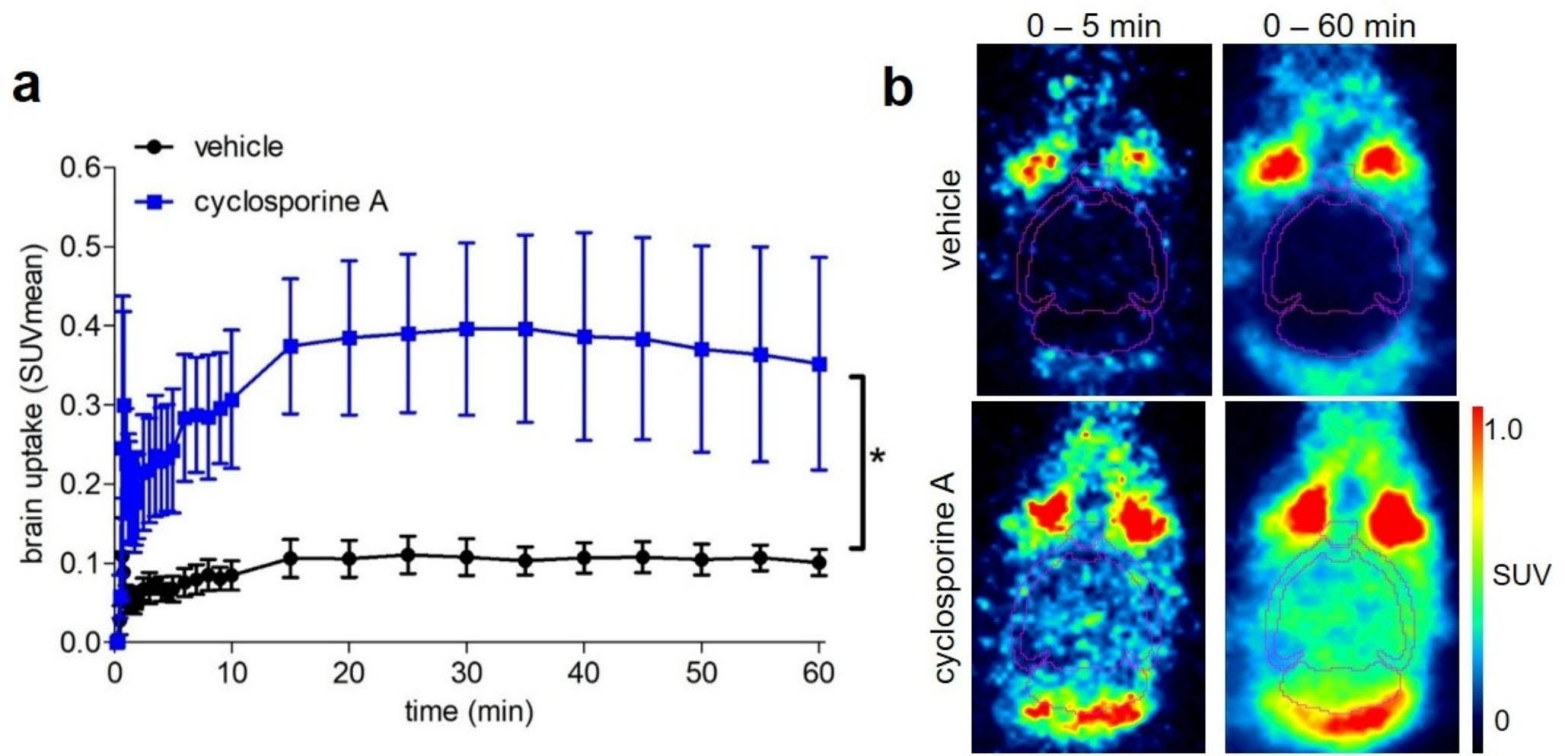
Efflux transporter substrate study in naïve CD-1 mice. (**a**) Time-activity curves of the brain (SUV_mean_) after pretreatment with vehicle (black dot; n = 4), or cyclosporine A (Sandimmune®, 50 mg/kg of cyclosporine A; blue square; n = 5). (**b**) Representative horizontal brain PET images. Student t-test: p < 0.0001*

To investigate the potential of [^18^F]**AG-120** for brain imaging, we performed a pilot PET study in CD-1 mice. The data showed a remarkable accumulation of activity only in the gallbladder, small intestine and urinary bladder indicating both urinary and hepatobiliary excretion, but a negligible uptake in the brain (Fig. S9, Tab. S3). We therefore treated the animals with cyclosporine A, an inhibitor of the P-glycoprotein (P-gp) efflux transporter expressed on the endothelial cells of the blood-brain barrier (BBB). Brain uptake was increased approximately 3-fold compared to vehicle, indicating that [^18^F]**AG-120** is a substrate of the P-gp (Fig. 6). Complementary post-mortem autoradiographic analysis of the activity distribution in the brain of a cyclosporine A pretreated mouse confirmed a homogeneous distribution pattern, indicating negligible binding of [^18^F]**AG-120** either to non-specific binding sites or to the evenly distributed IDH1 in the healthy brain (Fig. S11).

### Dynamic PET in rats bearing glioma xenografts

To evaluate the potential of [^18^F]**AG-120** to detect IDH1R312H-positive brain tumors in vivo, we implanted the transfected U251 cells stereotactically in nude rats to generate a suitable model of IDH1 and IDH1R132H-positive glioma (n = 2 for each cell type). Immunofluorescence staining of subsequently obtained brain sections and genome sequencing confirmed the presence of IDH1R132H in the mutant tumors (Fig. S5-S12a, Tab. S6).

**Fig. 7:**
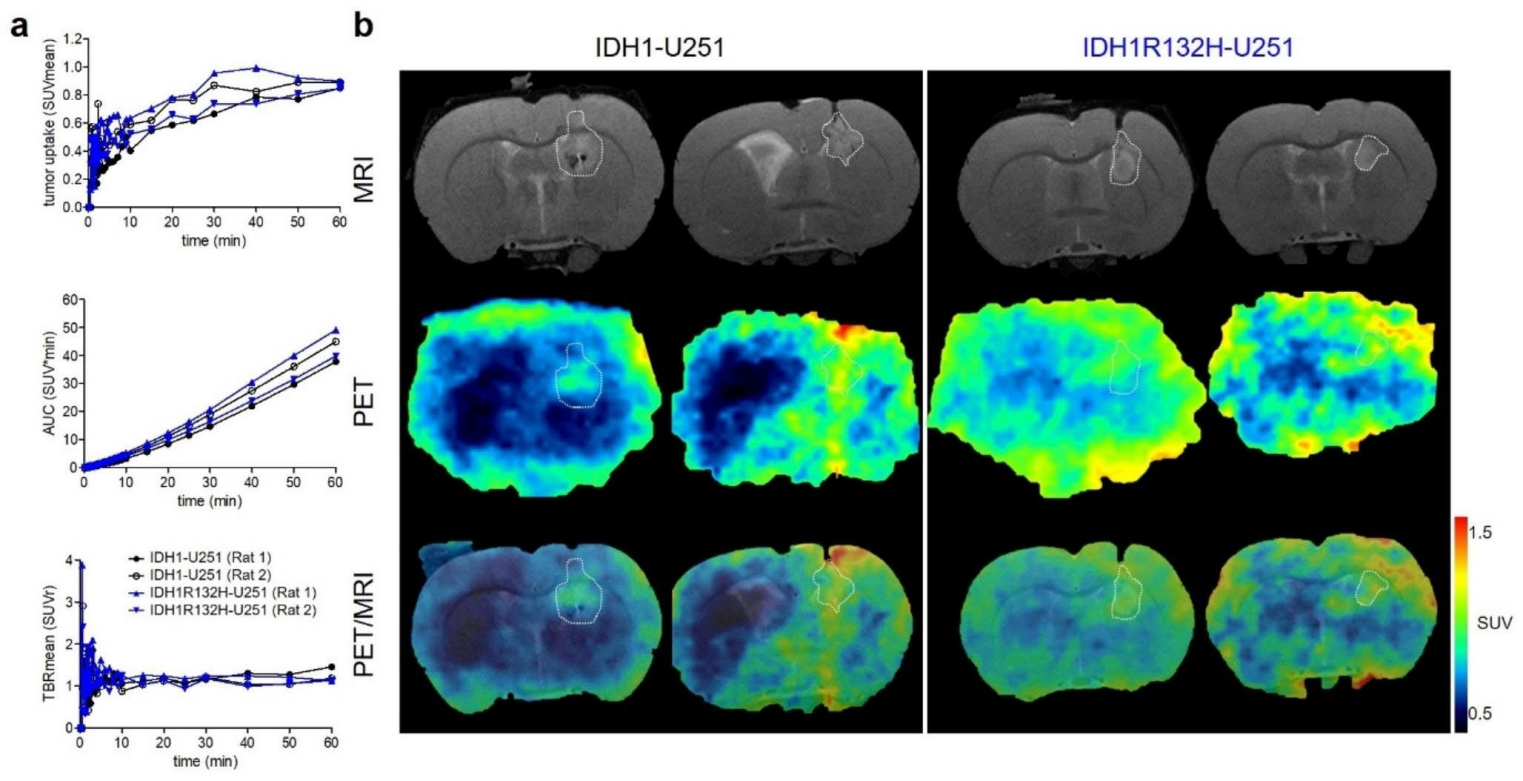
Tumor model PET study. (**a**) Time-activity curves of [^18^F]**FET** uptake respectively, in the tumor region of IDH1 (n = 2) and IDH1R132H tumor animals (n = 2). (**b**) Representative coronal multimodal images of the brain of IDH1 (n = 2) and IDH1R132H tumor animals (n = 2) of [^18^F]**FET** distribution respectively. PET images: 40–60 min time frame. MRI: T2-weighted images

Contrast-enhanced T1-weighted MR scans indicated a disruption of the BBB in both tumor models (Fig S12). To compare the cell density and proliferation of the two tumors, we first performed dynamic [^18^F]**FET**-PET scans to measure the correlating L-amino acid uptake in the IDH1-U251 and IDH1R132H-U251 glioma at 30 days after xenotransplantation [54]. Activity uptake was comparable in both tumor types, as shown by the TACs in Fig. 7 and by the subsequently extracted parameters time-to-peak, TAC-peak value, slope and AUC (Fig. 7a, Tab. S4). Similar TBR_mean_ values confirm a good comparability of the two glioma models in terms of density and proliferative capacity (Fig. 7b, Tab. S4).

**Fig. 8:**
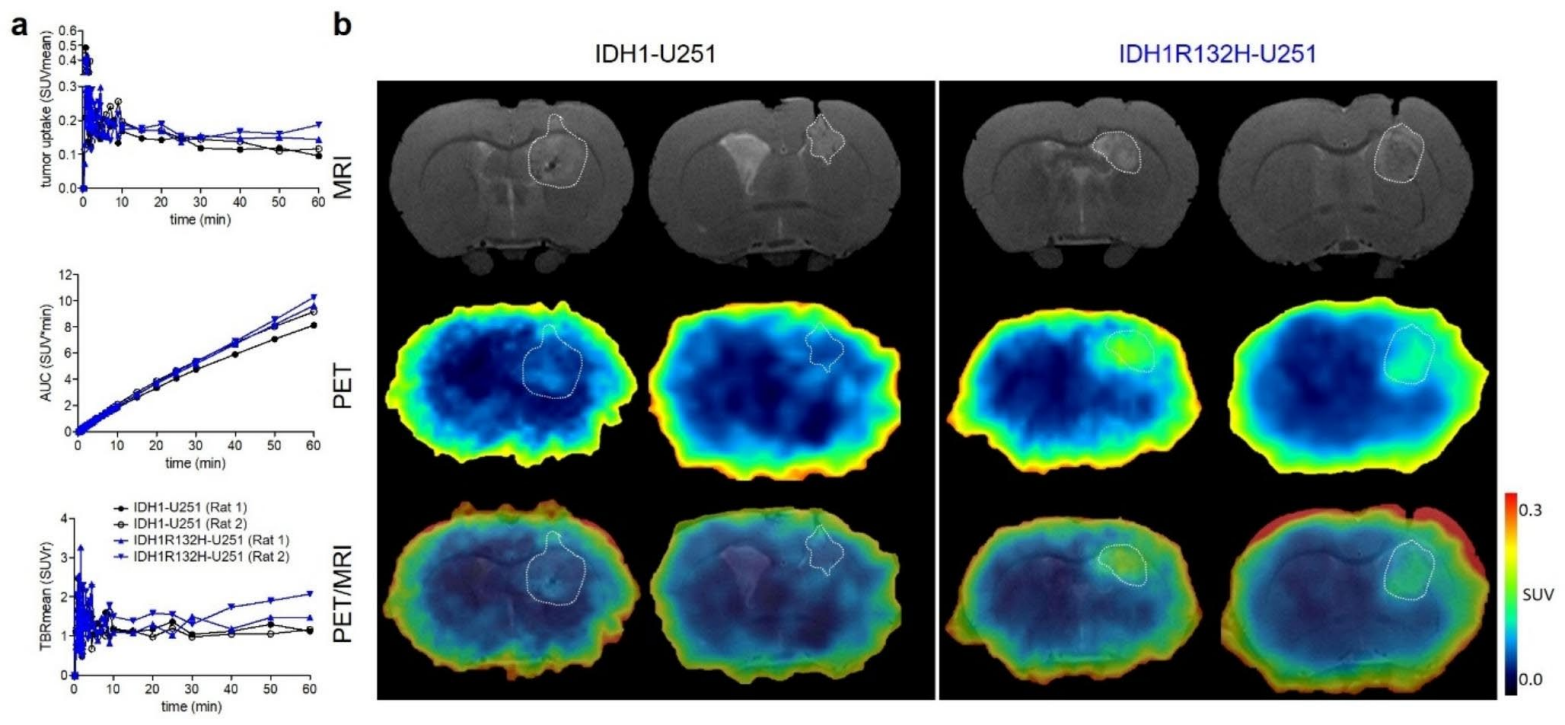
Tumor model PET study. (**A**) Time-activity curves of [^18^F]**AG-120** uptake, respectively, in the tumor region of IDH1 (n = 2) and IDH1R132H tumor animals (n = 2). (**B**) Representative coronal multimodal images of the brain of IDH1 (n = 2) and IDH1R132H tumor animals (n = 2) of [^18^F]**AG-120** distribution, respectively. PET images: 40–60 min time frame. MRI: T2-weighted images

Four days later, the animals were investigated by dynamic [^18^F]**AG-120** PET. Visual inspection of the TACs shows a similar initial uptake in the IDH1 and IDH1R132H tumor regions (TAC peak SUV_mean_: 0.46 vs. 0.41) with a slightly slower kinetics in the mutant tumor (time-to-peak: 0.75 vs. 1.17 min) (Fig. 8, Tab. S5). A slower washout of [^18^F]**AG-120** from the IDH1R132H tumor compared to the IDH1 tumor results in a slightly higher activity concentration at later time points (30–60 min p.i.), resulting in AUC_0–60 min_ values of 9.96 and 8.65 SUV*min, respectively (Fig. 8). However, the interpretation of the data is complicated by a potential non-specific accumulation of activity in the brain tumors of animals due to impairment of the BBB, which is indicated by a contrast enhancement of the tumors after i.v. injection of the contrast agent Gadovist^®^ (Fig. S12b).

## Discussion

To the best of our knowledge, this is the first study to assess the dynamics and strength of the physical interaction between the mIDH inhibitor [^18^F]**AG-120** and homodimers of IDH1 and IDH1R132H using radioligand binding. We observed a slow and apparently irreversible association of [^18^F]**AG-120** with a single binding site in the IDH1R132H-U251 cell lysates, which can be abolished by co-incubation with the structurally different mIDH inhibitor **BAY1436032**. Saturation analyses indicate an apparent affinity towards IDH1R132H in the low nanomolar range (*K*_D,app_~15 nM), corresponding to its inhibitory potency (IC_50_ ~ 4 nM). Parallel radioligand binding studies using preparations from IDH1- and IDH1R132H-transfected cells indicated a 4-fold higher binding potential of [^18^F]**AG-120** in the latter. Although these results indicate the suitability of [^18^F]**AG-120** for selective imaging of IDH1R132H, corresponding to the selective inhibition of the mutant enzyme, the results of our experiments also indicate a comparable apparent affinity of the radioligand to both enzymes in accordance with the results of Liu et al. [53]. However, clarification of the mechanism and implications of this finding was beyond the scope of this pilot study. The results of the cellular uptake studies were generally consistent with the binding results: Indeed, the uptake in the IDH1R132H-U251 cells is slower than in the IDH1-U251 cells but reaches a 28-fold higher concentration and can be blocked by pre-incubation with **BAY1436032** and appears to be irreversible.

In summary, our in vitro studies suggest that [^18^F]**AG-120** exhibits high affinity with slow association to IDH1R132H, as suggested by the initial characterization of **AG-120** as a slow binder [55], and apparently no dissociation. Whether the fact that [^18^F]**AG-120** occupies a much smaller fraction of binding sites in IDH1-U251 cells, albeit with an affinity comparable to that of the mutant enzyme, is sufficient for selective imaging of IDH1R132H remains to be investigated in future studies.

In vivo studies presented here confirmed the high metabolic stability of [^18^F]**AG-120** demonstrated in the human ADME study of Prakash et al. [37]. In addition, despite the fact that **AG-120** fulfills the theoretical requirements for a CNS radiotracer [56], the initial uptake of [^18^F]**AG-120** in the brain of naïve CD-1 mice was negligible with an SUV_mean_ of 0.1. The 3-fold higher brain uptake observed after pre-treatment with cyclosporine A suggests that the low penetration rate of **AG-120** was at least partly due to active P-gp-mediated efflux, as recently concluded from an in vitro study [57]. Nevertheless, based on the promising outcome of the cell uptake study, we decided to conduct a first pilot study to more specifically elucidate the potential of [^18^F]**AG-120** for tumor imaging. Accordingly, PET studies were performed using an orthotopic rat glioma model overexpressing the target or the off-target obtained by stereotactic implantation of the stably transfected IDH1R132H-U251 or IDH1-U251 cells, respectively. Due to the high-grade brain tumor origin of the implanted human U251 cells (glioblastoma), a disrupted BBB was expected, allowing the proof-of-concept to be performed independently of the BBB penetrance of [^18^F]**AG-120**. Limitations of this study related to the transfected U251 cell lines: (i) artificial overexpression of the IDH enzymes and (ii) impaired BBB integrity should be considered in the design of future studies.

The complex nature of tumors and their microenvironments characterized by longitudinal and spatial heterogeneity introduces various confounding factors that can influence the pharmacokinetics of a tumor-targeting radiotracer [58, 59]. Therefore, it is crucial to include suitable controls, such as the group of animals expressing the off-target. We intend to investigate the IDH1 and IDH1R132H tumor-bearing animals with comparable tumor size, homogeneous T2-weighted signal and positive contrast-enhanced T1-weighted MR images. An exploratory [^18^F]**FET**-PET study revealed similar uptake of the radiolabeled amino acid in both models. Despite the small sample size of our pilot study (two animals per group), the slightly higher TBR_max_ and TBR_mean_ values in the IDH1 compared to the IDH1R132H tumor align with clinical findings reported in the literature [33, 60, 61]. The results of our [^18^F]**AG-120** PET study indicate a comparable uptake of activity in both models with a slightly higher activity concentration observed in the IDH1R132H tumor at the end of the 1-h examination. However, due to the exploratory nature of the study and the very low activity concentrations in the target regions (SUVs ≤ 0.5) cautious interpretation is necessary to avoid over-evaluation. Despite this limitation, the specific trend of a slow but constant accumulation of activity in the IDH1R132H tumor in vivo is consistent with our in vitro findings and suggests that PET acquisitions of 2 h or even longer may reveal a more pronounced difference in activity uptake between IDH1R132H negative and positive tumors. Although evaluating the radiotracer in a patient-derived xenograft (PDX) model would provide valuable data on in vivo binding at pathophysiological levels of expression, IDH1R132H glioma PDX models are currently scarce, mainly due to their poor engraftment and slow growth rates.

## Conclusion

In conclusion, this study presents the radiosynthesis of stereoisomerically pure [^18^F]**AG-120** and provides valuable insights into its binding characteristics with IDH1 and IDH1R132H. [^18^F]**AG-120** will serve as a reference compound for future evaluation of mIDH inhibitors or radioligands and may have potential applications in peripheral tumors such as chondrosarcoma.

### Electronic supplementary material

Below is the link to the electronic supplementary material.

Supplementary Material 1

Supplementary Material 2

## Data Availability

The data sets generated during and/or analyzed during the current study are available from the corresponding author upon reasonable request.

## References

[1] Albert NL et al. ‘Response Assessment in Neuro-Oncology working group and European Association for Neuro-Oncology recommendations for the clinical use of PET imaging in gliomas’, *NEUONC*, vol. 18, no. 9, pp. 1199–1208, Sep. 2016, 10.1093/neuonc/now058. 10.1093/neuonc/now058 PMC4999003 27106405

[2] Jemal A et al. ‘Annual Report to the Nation on the Status of Cancer, 1975–2014, Featuring Survival’, *JNCI: Journal of the National Cancer Institute*, vol. 109, no. 9, Sep. 2017, 10.1093/jnci/djx030. 10.1093/jnci/djx030 PMC5409140 28376154

[3] Dejaegher J, Vleeschouwer SD. ‘Recurring Glioblastoma: A Case for Reoperation?’, in *Glioblastoma*, Department of Neurosurgery, University Hospitals Leuven, Leuven, Belgium and S. De Vleeschouwer, Eds., Codon Publications, 2017, pp. 281–296. 10.15586/codon.glioblastoma.2017.ch14. 29251867

[4] Weller M et al. ‘EANO guidelines on the diagnosis and treatment of diffuse gliomas of adulthood’, *Nat Rev Clin Oncol*, vol. 18, no. 3, pp. 170–186, Mar. 2021, 10.1038/s41571-020-00447-z. 10.1038/s41571-020-00447-z PMC7904519 33293629

[5] Miller JJ et al. ‘Accelerated progression of IDH mutant glioma after first recurrence’, *Neuro Oncol*, vol. 21, no. 5, pp. 669–677, May 2019, 10.1093/neuonc/noz016. 10.1093/neuonc/noz016 PMC6502499 30668823

[6] Yan H, et al. *IDH1* and *IDH2* mutations in Gliomas. N Engl J Med. Feb. 2009;360(8):765–73. 10.1056/NEJMoa0808710. 10.1056/NEJMoa0808710 PMC2820383 19228619

[7] Hartmann C et al. ‘Type and frequency of IDH1 and IDH2 mutations are related to astrocytic and oligodendroglial differentiation and age: a study of 1,010 diffuse gliomas’, *Acta Neuropathol*, vol. 118, no. 4, pp. 469–474, Oct. 2009, 10.1007/s00401-009-0561-9. 10.1007/s00401-009-0561-9 19554337

[8] Balss J, Meyer J, Mueller W, Korshunov A, Hartmann C, von Deimling A. ‘Analysis of the IDH1 codon 132 mutation in brain tumors’, *Acta Neuropathol*, vol. 116, no. 6, pp. 597–602, Dec. 2008, 10.1007/s00401-008-0455-2. 10.1007/s00401-008-0455-2 18985363

[9] Dang L et al. ‘Cancer-associated IDH1 mutations produce 2-hydroxyglutarate’, *Nature*, vol. 462, no. 7274, pp. 739–744, Dec. 2009, 10.1038/nature08617. 10.1038/nature08617 PMC2818760 19935646

[10] Juratli TA, Peitzsch M, Geiger K, Schackert G, Eisenhofer G, Krex D. ‘Accumulation of 2-hydroxyglutarate is not a biomarker for malignant progression in IDH-mutated low-grade gliomas’, *Neuro Oncol*, vol. 15, no. 6, pp. 682–690, Jun. 2013, 10.1093/neuonc/not006. 10.1093/neuonc/not006 PMC3661092 23410661

[11] Han S, et al. IDH mutation in glioma: molecular mechanisms and potential therapeutic targets. Br J Cancer. May 2020;122(11):1580–9. 10.1038/s41416-020-0814-x. 10.1038/s41416-020-0814-x PMC7250901 32291392

[12] Bardella C, et al. Expression of Idh1R132H in the murine Subventricular Zone Stem Cell Niche recapitulates features of early gliomagenesis. Cancer Cell. Oct. 2016;30(4):578–94. 10.1016/j.ccell.2016.08.017. 10.1016/j.ccell.2016.08.017 PMC5064912 27693047

[13] Sasaki M et al. ‘IDH1(R132H) mutation increases murine haematopoietic progenitors and alters epigenetics’, *Nature*, vol. 488, no. 7413, pp. 656–659, Aug. 2012, 10.1038/nature11323. 10.1038/nature11323 PMC4005896 22763442

[14] Pirozzi CJ, et al. Mutant IDH1 disrupts the mouse Subventricular Zone and alters Brain Tumor progression. Mol Cancer Res. May 2017;15(5):507–20. 10.1158/1541-7786.MCR-16-0485. 10.1158/1541-7786.MCR-16-0485 PMC5415422 28148827

[15] Rohle D, et al. An inhibitor of mutant IDH1 delays growth and promotes differentiation of Glioma cells. Science. May 2013;340(6132):626–30. 10.1126/science.1236062. 10.1126/science.1236062 PMC3985613 23558169

[16] Machida Y, et al. A potent blood–brain barrier-permeable mutant IDH1 inhibitor suppresses the growth of Glioblastoma with IDH1 mutation in a patient-derived Orthotopic Xenograft Model. Mol Cancer Ther. Feb. 2020;19(2):375–83. 10.1158/1535-7163.MCT-18-1349. 10.1158/1535-7163.MCT-18-1349 31727689

[17] Choi BD, Curry WT. IDH mutational status and the immune system in gliomas: a tale of two tumors? Transl Cancer Res. Oct. 2017;6:S1253–6. 10.21037/tcr.2017.09.37. 10.21037/tcr.2017.09.37 PMC5669623 29104858

[18] Louis DN et al. ‘The 2021 WHO Classification of Tumors of the Central Nervous System: a summary’, *Neuro-Oncology*, vol. 23, no. 8, pp. 1231–1251, Aug. 2021, 10.1093/neuonc/noab106. 10.1093/neuonc/noab106 PMC8328013 34185076

[19] Louis DN, et al. The 2016 World Health Organization Classification of Tumors of the Central Nervous System: a summary. Acta Neuropathol. Jun. 2016;131(6):803–20. 10.1007/s00401-016-1545-1. 10.1007/s00401-016-1545-1 27157931

[20] Mellinghoff IK et al. ‘Vorasidenib in IDH1- or IDH2-Mutant Low-Grade Glioma’, *N Engl J Med*, Jun. 2023, 10.1056/NEJMoa2304194. 10.1056/NEJMoa2304194 PMC11445763 37272516

[21] Platten M, et al. A vaccine targeting mutant IDH1 in newly diagnosed glioma. Nature. Apr. 2021;592(7854):463–8. 10.1038/s41586-021-03363-z. 10.1038/s41586-021-03363-z PMC8046668 33762734

[22] Mellinghoff IK et al. ‘Ivosidenib in Isocitrate Dehydrogenase 1 – Mutated Advanced Glioma’, *JCO*, vol. 38, no. 29, pp. 3398–3406, Oct. 2020, 10.1200/JCO.19.03327. 10.1200/JCO.19.03327 PMC7527160 32530764

[23] Mellinghoff IK, et al. Vorasidenib, a dual inhibitor of mutant IDH1/2, in recurrent or Progressive Glioma; results of a first-in-human phase I Trial. Clin Cancer Res. Aug. 2021;27(16):4491–9. 10.1158/1078-0432.CCR-21-0611. 10.1158/1078-0432.CCR-21-0611 PMC8364866 34078652

[24] Neumaier F, Zlatopolskiy BD, Neumaier B. ‘Mutated Isocitrate Dehydrogenase (mIDH) as Target for PET Imaging in Gliomas’, *Molecules*, vol. 28, no. 7, p. 2890, Mar. 2023, 10.3390/molecules28072890. 10.3390/molecules28072890 PMC10096429 37049661

[25] Jafari-Khouzani K, et al. Volumetric relationship between 2-hydroxyglutarate and FLAIR hyperintensity has potential implications for radiotherapy planning of mutant *IDH* glioma patients. Neuro Oncol. Jul. 2016;100:now. 10.1093/neuonc/now100. 10.1093/neuonc/now100 PMC5063518 27382115

[26] Verger A, et al. IDH mutation is paradoxically associated with higher 18F-FDOPA PET uptake in diffuse grade II and grade III gliomas. Eur J Nucl Med Mol Imaging. Aug. 2017;44(8):1306–11. 10.1007/s00259-017-3668-6. 10.1007/s00259-017-3668-6 28293705

[27] Cicone F, et al. 18F-DOPA uptake does not correlate with IDH mutation status and 1p/19q co-deletion in glioma. Ann Nucl Med. Apr. 2019;33(4):295–302. 10.1007/s12149-018-01328-3. 10.1007/s12149-018-01328-3 30607877

[28] Blanc-Durand P et al. ‘Voxel-based 18F-FET PET segmentation and automatic clustering of tumor voxels: A significant association with IDH1 mutation status and survival in patients with gliomas’, *PLoS ONE*, vol. 13, no. 6, p. e0199379, Jun. 2018, 10.1371/journal.pone.0199379. 10.1371/journal.pone.0199379 PMC6023198 29953478

[29] Wollring MM, et al. Clinical applications and prospects of PET imaging in patients with IDH-mutant gliomas. J Neurooncol. Dec. 2022. 10.1007/s11060-022-04218-x. 10.1007/s11060-022-04218-x PMC10227162 36577872

[30] Zaragori T, Guedj E, Verger A. ‘Is IDH mutation status associated with 18F-FDopa PET uptake?’, *Ann Nucl Med*, vol. 34, no. 3, pp. 228–229, Mar. 2020, 10.1007/s12149-020-01442-1. 10.1007/s12149-020-01442-1 32002736

[31] Suchorska B et al. ‘Identification of time-to-peak on dynamic 18F-FET-PET as a prognostic marker specifically in IDH1/2 mutant diffuse astrocytoma’, *Neuro-Oncology*, vol. 20, no. 2, pp. 279–288, Jan. 2018, 10.1093/neuonc/nox153. 10.1093/neuonc/nox153 PMC5777500 29016996

[32] Unterrainer M et al. ‘Serial ^18^ F-FET PET Imaging of Primarily ^18^ F-FET–Negative Glioma: Does It Make Sense?’, *J Nucl Med*, vol. 57, no. 8, pp. 1177–1182, Aug. 2016, 10.2967/jnumed.115.171033. 10.2967/jnumed.115.171033 27033893

[33] Unterrainer M et al. ‘Comparison of 18F-GE-180 and dynamic 18F-FET PET in high grade glioma: a double-tracer pilot study’, *Eur J Nucl Med Mol Imaging*, vol. 46, no. 3, pp. 580–590, Mar. 2019, 10.1007/s00259-018-4166-1. 10.1007/s00259-018-4166-1 30244386

[34] Verger A, Imbert L, Zaragori T. ‘Dynamic amino-acid PET in neuro-oncology: a prognostic tool becomes essential’, *Eur J Nucl Med Mol Imaging*, vol. 48, no. 13, pp. 4129–4132, Dec. 2021, 10.1007/s00259-021-05530-w. 10.1007/s00259-021-05530-w 34518904

[35] Miller JJ et al. ‘Isocitrate dehydrogenase (IDH) mutant gliomas: A Society for Neuro-Oncology (SNO) consensus review on diagnosis, management, and future directions’, *Neuro-Oncology*, vol. 25, no. 1, pp. 4–25, Jan. 2023, 10.1093/neuonc/noac207. 10.1093/neuonc/noac207 PMC9825337 36239925

[36] Obara T, et al. Adult diffuse low-Grade gliomas: 35-Year experience at the Nancy France Neurooncology Unit. Front Oncol. Oct. 2020;10:574679. 10.3389/fonc.2020.574679. 10.3389/fonc.2020.574679 PMC7656991 33194684

[37] Prakash C, Fan B, Altaf S, Agresta S, Liu H, Yang H. Pharmacokinetics, absorption, metabolism, and excretion of [14 C]ivosidenib (AG-120) in healthy male subjects. Cancer Chemother Pharmacol. May 2019;83(5):837–48. 10.1007/s00280-019-03793-7. 10.1007/s00280-019-03793-7 30758648

[38] Weber V et al. ‘Novel Radioiodinated and Radiofluorinated Analogues of FT-2102 for SPECT or PET Imaging of mIDH1 Mutant Tumours’, *Molecules*, vol. 27, no. 12, p. 3766, Jun. 2022, 10.3390/molecules27123766. 10.3390/molecules27123766 PMC9228733 35744895

[39] Chitneni SK, Yan H, Zalutsky MR. ‘Synthesis and Evaluation of a ^18^ F-Labeled Triazinediamine Analogue for Imaging Mutant IDH1 Expression in Gliomas by PET’, *ACS Med. Chem. Lett*, vol. 9, no. 7, pp. 606–611, Jul. 2018, 10.1021/acsmedchemlett.7b00478. 10.1021/acsmedchemlett.7b00478 PMC6047021 30034587

[40] Wang T, et al. Synthesis and biological evaluation of novel PET tracers [18F]AG120 & [18F]AG135 for imaging mutant isocitrate dehydrogenase 1 expression. Bioorg Med Chem. Jan. 2022;53:116525. 10.1016/j.bmc.2021.116525. 10.1016/j.bmc.2021.116525 34871844

[41] Kessler J et al. ‘IDH1(R132H) mutation causes a less aggressive phenotype and radiosensitizes human malignant glioma cells independent of the oxygenation status’, *Radiother Oncol*, vol. 116, no. 3, pp. 381–387, Sep. 2015, 10.1016/j.radonc.2015.08.007. 10.1016/j.radonc.2015.08.007 26328938

[42] Zarrad F, Zlatopolskiy B, Krapf P, Zischler J, Neumaier B. A practical method for the Preparation of 18F-Labeled aromatic amino acids from nucleophilic [18F]fluoride and stannyl precursors for Electrophilic Radiohalogenation. Molecules. Dec. 2017;22(12):2231. 10.3390/molecules22122231. 10.3390/molecules22122231 PMC6149761 29244780

[43] Makaravage KJ, Brooks AF, Mossine AV, Sanford MS, Scott PJH. Copper-mediated Radiofluorination of arylstannanes with [ ^18^ F]KF. Org Lett. Oct. 2016;18(20):5440–3. 10.1021/acs.orglett.6b02911. 10.1021/acs.orglett.6b02911 PMC5078836 27718581

[44] Tago T, Toyohara J, Ishii K. ‘Preclinical Evaluation of an ^18^ F-Labeled SW-100 Derivative for PET Imaging of Histone Deacetylase 6 in the Brain’, *ACS Chem. Neurosci*, vol. 12, no. 4, pp. 746–755, Feb. 2021, 10.1021/acschemneuro.0c00774. 10.1021/acschemneuro.0c00774 33502174

[45] Bowden GD, Chailanggar N, Pichler BJ, Maurer A (2021). Scalable ^18^ F processing conditions for copper-mediated radiofluorination chemistry facilitate DoE optimization studies and afford an improved synthesis of [ ^18^ F]olaparib. Org Biomol Chem.

[46] Guibbal F, et al. Manual and automated Cu-mediated radiosynthesis of the PARP inhibitor [18F]olaparib. Nat Protoc. Apr. 2020;15(4):1525–41. 10.1038/s41596-020-0295-7. 10.1038/s41596-020-0295-7 32111986

[47] Antuganov D et al. ‘Copper-Mediated Radiofluorination of Aryl Pinacolboronate Esters: A Straightforward Protocol by Using Pyridinium Sulfonates’, *Eur J Org Chem*, vol. 2019, no. 5, pp. 918–922, Feb. 2019, 10.1002/ejoc.201801514.

[48] Zhang X, Basuli F, Swenson RE. ‘An azeotropic drying-free approach for copper-mediated radiofluorination without addition of base’, *J Label Compd Radiopharm*, vol. 62, no. 3, pp. 139–145, Mar. 2019, 10.1002/jlcr.3705. 10.1002/jlcr.3705 PMC6428597 30644121

[49] Mossine AV, Brooks AF, Ichiishi N, Makaravage KJ, Sanford MS, Scott PJH. Sci Rep. Mar. 2017;7(1):233. 10.1038/s41598-017-00110-1. ‘Development of Customized [18F]Fluoride ElutionTechniques for the Enhancement of Copper-Mediated Late-Stage Radiofluorination’. 10.1038/s41598-017-00110-1 PMC5427906 28331174

[50] Wright JS et al. ‘Copper-mediated late-stage radiofluorination: five years of impact on preclinical and clinical PET imaging’, *Clin Transl Imaging*, vol. 8, no. 3, pp. 167–206, Jun. 2020, 10.1007/s40336-020-00368-y. 10.1007/s40336-020-00368-y PMC7968072 33748018

[51] Xu X et al. ‘Structures of Human Cytosolic NADP-dependent Isocitrate Dehydrogenase Reveal a Novel Self-regulatory Mechanism of Activity’, *Journal of Biological Chemistry*, vol. 279, no. 32, pp. 33946–33957, Aug. 2004, 10.1074/jbc.M404298200. 10.1074/jbc.M404298200 15173171

[52] Liu S et al. ‘Roles of metal ions in the selective inhibition of oncogenic variants of isocitrate dehydrogenase 1’, *Commun Biol*, vol. 4, no. 1, p. 1243, Nov. 2021, 10.1038/s42003-021-02743-5. 10.1038/s42003-021-02743-5 PMC8560763 34725432

[53] Liu S, Abboud M, Mikhailov V, Liu X, Reinbold R, Schofield CJ. ‘Differentiating Inhibition Selectivity and Binding Affinity of Isocitrate Dehydrogenase 1 Variant Inhibitors’, *J. Med. Chem*, vol. 66, no. 7, pp. 5279–5288, Apr. 2023, 10.1021/acs.jmedchem.3c00203. 10.1021/acs.jmedchem.3c00203 PMC10108345 36952395

[54] Hutterer M et al. ‘[18F]-fluoro-ethyl-l-tyrosine PET: a valuable diagnostic tool in neuro-oncology, but not all that glitters is glioma’, *Neuro-Oncology*, vol. 15, no. 3, pp. 341–351, Mar. 2013, 10.1093/neuonc/nos300. 10.1093/neuonc/nos300 PMC3578481 23335162

[55] Popovici-Muller J et al. ‘Discovery of AG-120 (Ivosidenib): A First-in-Class Mutant IDH1 Inhibitor for the Treatment of IDH1 Mutant Cancers’, *ACS Med. Chem. Lett*, vol. 9, no. 4, pp. 300–305, Apr. 2018, 10.1021/acsmedchemlett.7b00421. 10.1021/acsmedchemlett.7b00421 PMC5900343 29670690

[56] Lipinski CA, Lombardo F, Dominy BW, Feeney PJ. ‘Experimental and computational approaches to estimate solubility and permeability in drug discovery and development settings 1PII of original article: S0169-409X(96)00423-1. The article was originally published in Advanced Drug Delivery Reviews 23 (1997) 3–25. 1’, *Advanced Drug Delivery Reviews*, vol. 46, no. 1–3, pp. 3–26, Mar. 2001, 10.1016/S0169-409X(00)00129-0. 10.1016/s0169-409x(00)00129-0 11259830

[57] Urban DJ, et al. Assessing inhibitors of mutant isocitrate dehydrogenase using a suite of pre-clinical discovery assays. Sci Rep. Oct. 2017;7(1):12758. 10.1038/s41598-017-12630-x. 10.1038/s41598-017-12630-x PMC5630632 28986582

[58] Viel T et al. ‘Analysis of the Growth Dynamics of Angiogenesis-Dependent and -Independent Experimental Glioblastomas by Multimodal Small-Animal PET and MRI’, *J Nucl Med*, vol. 53, no. 7, pp. 1135–1145, Jul. 2012, 10.2967/jnumed.111.101659. 10.2967/jnumed.111.101659 22689925

[59] Stegmayr C et al. ‘Influence of blood-brain barrier permeability on O-(2-18F-fluoroethyl)-L-tyrosine uptake in rat gliomas’, *Eur J Nucl Med Mol Imaging*, vol. 44, no. 3, pp. 408–416, Mar. 2017, 10.1007/s00259-016-3508-0. 10.1007/s00259-016-3508-0 27613541

[60] Verger A, et al. Static and dynamic 18F–FET PET for the characterization of gliomas defined by IDH and 1p/19q status. Eur J Nucl Med Mol Imaging. Mar. 2018;45(3):443–51. 10.1007/s00259-017-3846-6. 10.1007/s00259-017-3846-6 29043400

[61] Vettermann F et al. ‘Non-invasive prediction of IDH-wildtype genotype in gliomas using dynamic 18F-FET PET’, *Eur J Nucl Med Mol Imaging*, vol. 46, no. 12, pp. 2581–2589, Nov. 2019, 10.1007/s00259-019-04477-3. 10.1007/s00259-019-04477-3 31410540

