# Correction to: Radiosynthesis and biological evaluation of [^18^F]AG-120 for PET imaging of the mutant isocitrate dehydrogenase 1 in glioma

**DOI:** 10.1007/s00259-024-06614-z

**Published:** 2024-01-22

**Authors:** Thu Hang Lai, Barbara Wenzel, Sladjana Dukić-Stefanović, Rodrigo Teodoro, Lucie Arnaud, Aurélie Maisonial-Besset, Valérie Weber, Rareş-Petru Moldovan, Sebastian Meister, Jens Pietzsch, Klaus Kopka, Tareq A. Juratli, Winnie Deuther-Conrad, Magali Toussaint

**Affiliations:** 1 Institute of Radiopharmaceutical Cancer Research, Department of Neuroradiopharmaceuticals, Helmholtz-Zentrum Dresden-Rossendorf, Research site Leipzig, Leipzig, Germany; 2 Department of Research and Development, ROTOP Pharmaka GmbH, Dresden, Germany; 3 Université Clermont Auvergne, Imagerie Moléculaire et Stratégies Théranostiques, UMR 1240, Inserm, Clermont-Ferrand, France; 4 Institute of Radiopharmaceutical Cancer Research, Department of Radiopharmaceutical and Chemical Biology, Helmholtz-Zentrum Dresden-Rossendorf, Dresden, Germany; 5 School of Science, Faculty of Chemistry and Food Chemistry, Technische Universität Dresden, Dresden, Germany; 6 German Cancer Consortium (DKTK), Partner Site Dresden, Dresden, Germany; 7 National Center for Tumor Diseases (NCT) Dresden, University Hospital Carl Gustav Carus, Dresden, Germany; 8 Department of Neurosurgery, Faculty of Medicine, University Hospital Carl Gustav Carus, Technische Universität Dresden, Dresden, Germany

**Correction to: European Journal of Nuclear Medicine and Molecular Imaging**

https://doi.org/ 10.1007/s00259-023-06515-7

The authors regret that the version of Scheme 1 that appears in the original article is incorrect. The correct Scheme 1 is shown below.

**Scheme 1:**
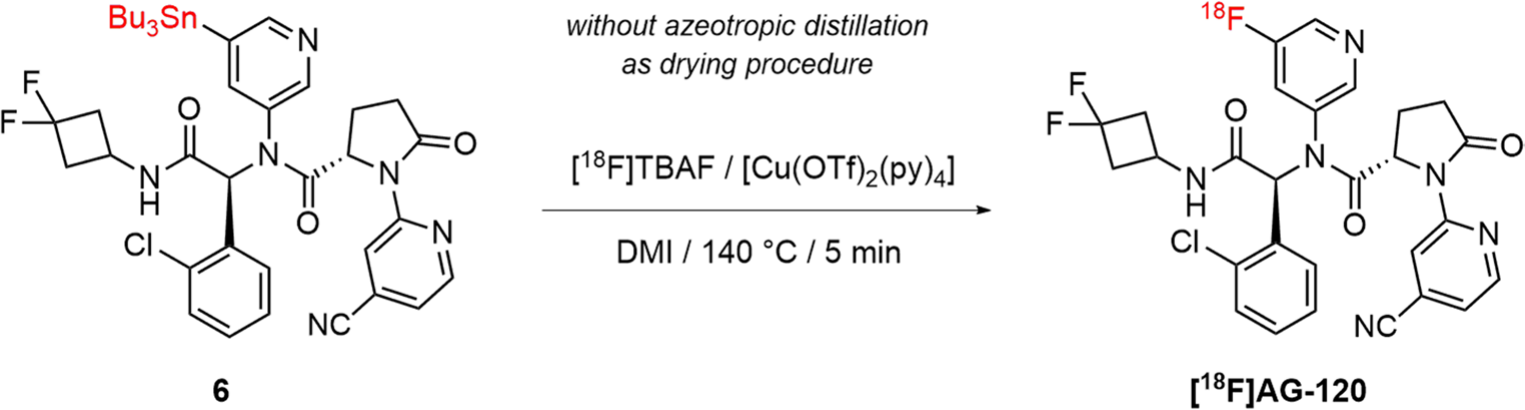
Copper-mediated radiofluorination of [^18^F]AG-120

The original article has been corrected.

