# Supplementary material for: Radiosynthesis and biological evaluation of [18F]AG-120 for PET imaging of the mutant isocitrate dehydrogenase 1 in glioma
Source: Eur J Nucl Med Mol Imaging. 2023 Nov 20;51(4):1085–96. doi: 10.1007/s00259-023-06515-7 (PMC10881675; doi:10.1007/s00259-023-06515-7)
Supplement: Supplementary file 2 — Supplementary Material 2 [file 259_2023_6515_MOESM2_ESM.pdf]

Supplementary Information: Radiosynthesis and biological evaluation of [^{18}F]AG-120 for PET imaging of the mutant isocitrate dehydrogenase 1 in glioma

Thu Hang Lai[1,2‡], Barbara Wenzel[1‡], Sladjana Dukić-Stefanović[1], Rodrigo Teodoro[1], Lucie Arnaud[3], Aurélie Maisonial-Besset[3], Valérie Weber[3], Rareş-Petru Moldovan[1], Sebastian Meister[4], Jens Pietzsch[4,5], Klaus Kopka[1,5,6,7], Tareq A. Juratli[7,8], Winnie Deuther-Conrad[1‡], Magali Toussaint[1‡*]

[1]*Helmholtz-Zentrum Dresden-Rossendorf, Institute of Radiopharmaceutical Cancer Research, Department of Neuroradiopharmaceuticals, Research site Leipzig, Germany*

[2]*ROTOP Pharmaka GmbH, Department of Research and Development, Dresden, Germany*

[3]*Université Clermont Auvergne, Inserm, Imagerie Moléculaire et Stratégies Théranostiques, UMR 1240, Clermont-Ferrand, France*

[4]*Helmholtz-Zentrum Dresden-Rossendorf, Institute of Radiopharmaceutical Cancer Research, Department of Radiopharmaceutical and Chemical Biology, Dresden, Germany*

[5]*Technische Universität Dresden, School of Science, Faculty of Chemistry and Food Chemistry, Dresden, Germany*

[6]*German Cancer Consortium (DKTK), Partner Site Dresden, Dresden, Germany*

[7]*National Center for Tumor Diseases (NCT) Dresden, University Hospital Carl Gustav Carus, Dresden, Germany*

[8]*Department of Neurosurgery, Faculty of Medicine and University Hospital Carl Gustav Carus, Technische Universität Dresden, Dresden, Germany.*

[‡]These authors contributed equally to this work

[*]**Corresponding authors:**

M.T., m.toussaint@hzdr.de, +49 341 234179 4616

Organic Chemistry 4
General 4
Chemical synthesis of the stannyl precursor **6** 4
Chemical synthesis of the diastereomeric mixture of (*S*,*R*)-**AG-120** and (*S*,*S*)-**AG-120** 7
Radiochemistry........ 8
General 8
Radiosynthesis of [^{18}F]**FET** 8
Fig. S1 9
Radiosynthesis of [^{18}F]**AG-120**........ 10
Tab. S1........ 11
Fig. S2 13
Fig. S3 14
Fig. S4 14
Biological evaluation........ 15
General 15
Cell culture 15
Immunofluorescence 15
Western Blotting........ 16
IDH1 enzyme assay for determination of inhibitory potency 16
Mutant IDH1 enzyme assay for determination of inhibitory potency 16
In vitro binding assays using [^{18}F]**AG-120** and lysates of stably transfected U251 cells 17
In vitro cell uptake studies using [^{18}F]**AG-120** and stably transfected U251 cells 18
In vivo evaluation – General 18
In vivo metabolism in mice 19
Ex vivo P-glycoprotein efflux transporter study 19
Dynamic PET studies in naïve mice........ 20
Orthotopic glioma model........ 20
Next-Generation Sequencing........ 21
MRI monitoring........ 21
Dynamic PET studies in orthotopic brain tumor model 22
Fig. S5 23
Fig. S6 24
Tab. S2........ 24
Fig S7 25
Tab. S3........ 26
Fig. S9 28
Fig. S10 28

Fig. S11 28
Fig. S12 29
Fig. S13 30
Tab. S4.. 30
Tab. S5.. 31

Organic Chemistry

General

All chemicals and reagents for the precursor synthesis of [^{18}F]**AG-120** (((*S*)-*N*-((*S*)-1-(2-chlorophenyl)-2-((3,3-difluorocyclobutyl)amino)-2-oxoethyl)-1-(5-cyanopyridin-2-yl)-*N*-(5-[^{18}F]fluoropyridin-3-yl)-5-oxopyrrolidine-2-carboxamide)) were purchased from commercially available sources and used without further purification. The synthesis was performed as described in literature with minor modifications [1]. The purity was determined by LC-MS [Dionex Ultimate 3000 system incorporating a LPG-3400SD pump, a WPS-3000 TSL autosampler, a TCC-3000SD column compartment, a DAD3000 diode array detector and a MSQ 3000 low-resolution mass spectrometer (Thermo Fisher Scientific Inc., Waltham; USA), column: Reprosil-Pur 120 C18-AQ (150×3 mm, 3 µm, Dr. Maisch GmbH, Ammerbuch, Germany), eluent mixture of MeCN/20 mM $NH_4OAc_{aq.}$ (pH 6.8) in gradient mode A (0–2.5 min 10% MeCN, 2.5-9 min up to 90% MeCN, 9-13 min 90% MeCN, 13-13.5 min up to 10% MeCN, 13.5-15 min 10% MeCN) at a flow rate of 0.6 mL/min, gradient mode B (0-1.5 min 5% MeCN, 1.5-8 min up to 85% MeCN, 8-12 min 85% MeCN, 12-12.5 min up to 5% MeCN, 12.5-15 min 5% MeCN) at a flow rate of 0.7 mL/min or isocratic mode (80% MeCN) at a flow rate of 0.8 mL/min. ^{1}H-, ^{13}C- and ^{19}F-NMR spectra were recorded on a BRUKER DRX-400 (400 MHz for ^{1}H-NMR, 101 MHz for ^{13}C-NMR, 377 MHz for ^{19}F-NMR). All spectra were recorded at room temperature (RT) followed by calibration on the solvent signal [$CDCl_3$: δ(^{1}H-NMR) = 7.26 ppm and δ(^{13}C-NMR) = 77.16 ppm]. High-resolution mass spectrometry (HRMS) was conducted in positive ion mode using an ESI-TOF microTOF instrument from Bruker Daltonik GmbH (Bremen, Germany). The simulation of mass spectra was carried out with the Web-based MS online tool of Scientific Instrument Services (SISweb, Palmer, MA). Analysis of NMR and MS data was performed using MestReNova version 12.0.0. Analytical thin-layer chromatography (TLC) was performed on silica gel coated plates (Macherey-Nagel, ALUGRAM SIL G/UV_{254}). The spots were detected by using an UV lamp or by dipping the plates into a $KMnO_4$-solution (3 g $KMnO_4$, 20 g K_2CO_3, 0.25 mL glacial acid, 300 mL water). Purification of the final products was assessed by flash column chromatography using silica gel 40–63 µm from VWR Chemicals.

Chemical synthesis of the stannyl precursor 6

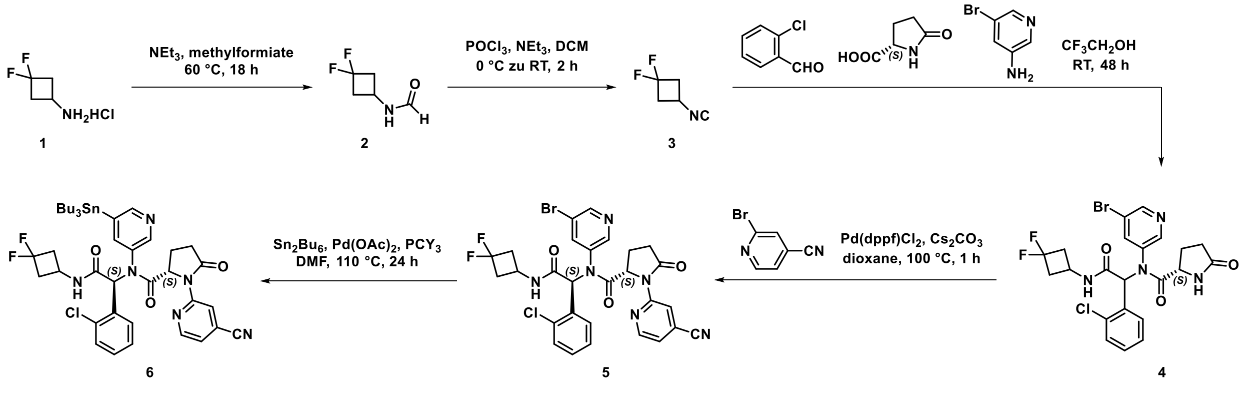

N-(3,3-difluorocyclobutyl)formamide (**2**): A pressure flask was charged with 3,3-difluorocyclobutanamine hydrochloride (**1**, 1 g, 7.0 mmol, 1 eq), methylformiate (8.6 mL, 139.9 mmol, 20 eq) and triethylamine (1.9 mL, 13.9 mmol, 2 eq). Then, the reaction mixture was heated at 50 °C overnight (TLC/SiO_2 in ethyl acetate, R_f = 0.56). After cooling to RT, the mixture was filtrated, washed with ethyl acetate (3 x 100 mL) and the combined filtrate was concentrated under reduced pressure. The residue was dissolved in ethyl acetate (300 mL), filtrated and the filtrate was concentrated under reduced pressure. The residue was washed with *n*-hexane (3 x 50 mL) and dried in vacuum. The desired product **2** was obtained as white solid (quantitative yield). ^{1}H-NMR (400 MHz, $CDCl_3$): δ = 8.13 (s, 1H), 6.15 (s, 1H), 4.45 – 4.22 (m, 1H), 3.08 – 2.90 (m, 2H), 2.60 – 2.40 (m, 2H). ^{19}F-NMR (377 MHz, $CDCl_3$): δ = -85.17 (ddddd, *J* = 199.3, 21.6, 13.3, 8.2, 5.0 Hz), -96.88 (ddddd, *J* = 199.3, 25.9, 14.6, 11.8, 3.2 Hz). ^{13}C-NMR (101 MHz, $CDCl_3$): δ = 161.12, 118.60 (dd, *J* = 280.1, 273.0 Hz), 43.58 – 42.72 (m), 33.83 (dd, *J* = 16.1, 8.1 Hz).

1,1-Difluoro-3-isocyanocyclobutane (**3**): To a solution of *N*-(3,3-difluorocyclobutyl)formamide (**2**, 100 mg, 0.7 mmol, 1 eq) in CH_2Cl_2 (5 mL) was added triethylamine (410 µL, 3.0 mmol, 4 eq). The mixture was cooled to 0 °C and a solution of phosphoryl chloride (76 µL, 0.8 mmol, 1.1 eq) in CH_2Cl_2 (1 mL) was added dropwise. The mixture was stirred at RT for 2 h (TLC/SiO_2 in ethyl acetate, R_f = 0-0.17) and then, concentrated under reduced pressure (RT, max. 50 mbar). The residue was dissolved in diethyl ether (100 mL), filtrated over silica and washed with diethyl ether (200 mL). After concentration under reduced pressure (RT, max. 50 mbar), the desired product **3** was obtained as yellow oil (76% yield, 63% purity). ^{1}H-NMR (400 MHz, $CDCl_3$): δ = 3.97 (pd, *J* = 7.3, 1.0 Hz, 1H), 3.17 – 3.02 (m, 2H), 3.01 – 2.78 (m, 2H). ^{19}F-NMR (377 MHz, $CDCl_3$): δ = -84.88 (ddddd, *J* = 201.1, 19.1, 12.1, 6.9, 4.5 Hz), -96.69 (dp, *J* = 201.0, 13.2 Hz). ^{13}C-NMR (101 MHz, $CDCl_3$): δ = 160.23 – 157.63 (m), 121.32 – 111.84 (m), 44.25 (t, *J* = 24.8 Hz), 35.35 (dq, *J* = 16.3, 7.9 Hz).

(2*S*)-*N*-(5-bromopyridin-3-yl)-*N*-(1-(2-chlorophenyl)-2-((3,3-difluorocyclobutyl)amino)-2-oxoethyl)-5-oxopyrrolidine-2-carboxamide (**4**): A mixture of 2-chlorobenzaldehyde (120 mg, 0.9 mmol, 1 eq), 5-bromopyridin-3-amine (148 mg, 0.9 mmol, 1 eq) in 2-fluoroethanol (2 mL) was stirred at RT for 30 min with molecular sieve. Then, (*S*)-5-oxopyrrolidine-2-carboxylic acid (168 mg, 0.9 mmol, 1 eq) was added and the mixture was stirred again at RT for 15 min. After addition of 1,1-difluoro-3-isocyanocyclobutane (**3**, 100 mg, 0.9 mmol, 1 eq), the reaction mixture was stirred at RT for 2 days (TLC/RP-Silica in MeOH/H_2O, 1/1, *v/v*, R_f = 0.36). The mixture was concentrated under reduced pressure and the residue was purified by flash chromatography (RP-silica, gradient MeOH/H_2O 1/1→ 1.5/1 → 2/1 → 1/1, *v/v*) to afford the desired product **4** as white solid (79% yield, mixture of two diastereomers). ^{1}H-NMR (400 MHz, $CDCl_3$): δ = 8.47 (d, *J* = 14.3 Hz, 4H), 7.41 (d, *J* = 8.1 Hz, 2H), 7.32 (d, *J* = 8.0 Hz, 2H), 7.25 – 7.15 (m, 2H), 7.10 – 6.77 (m, 6H), 6.58 – 6.39 (m, 4H), 4.39 – 4.00 (m, 4H), 3.13 – 2.77 (m, 4H), 2.66 – 1.81 (m, 12H). LC-MS (gradient mode A): purity = 95%, t_R = 9.4 min, m/z (ESI^+) = 558.10/560.11

(calcd. 558.07/560.07 for $[M+NH_4]^+$ with $^{79}Br/^{81}Br$), m/z (ESI^-) = 539.07/541.12 (calcd. 539.03/541.03 for $[M-H]^-$ with $^{79}Br/^{81}Br$).

(*S*)-*N*-(5-bromopyridin-3-yl)-*N*-((*S*)-1-(2-chlorophenyl)-2-((3,3-difluorocyclobutyl)amino)-2-oxoethyl)-1-(4-cyanopyridin-2-yl)-5-oxopyrrolidine-2-carboxamide (**5**): A solution of the diastereomeric mixture **4** (140 mg, 0.3 mmol, 1 eq), 2-bromoisonicotinonitrile (57 mg, 0.3 mmol, 1.2 eq), caesium carbonate (101 mg, 0.3 mmol, 1.2 eq), 4,5-bis(diphenylphosphino)-9,9-dimethylxanthene (Xantphos, 8 mg, 0.01 mmol, 0.05 eq) and [1,1′-bis(diphenylphosphino)ferrocene]dichloropalladium(II) (9 mg, 0.01 mmol, 0.05 eq) in dioxane (2 mL) was stirred at 100 °C for 1 h (TLC/SiO_2 in ethyl acetate/petrolether, 1/1, *v/v*, R_f = 0.45 and 0.35). After concentration of the mixture under pressure, the residue was dissolved in ethyl acetate (50 mL), washed with water (3 x 20 mL) and brine (20 mL), dried over anhydrous $MgSO_4$, filtrated and concentrated under reduced pressure. Then, the diastereomeric residue was purified by flash chromatography (silica, gradient ethyl acetate/PE, 1/3 → 1/2 → 1/1 → 2/1, *v/v*) to afford a mixture of two diastereomers). The diastereomers were separated by flash chromatography (silica, gradient CH_2Cl_2/EtOH 95/5 → 60/40, *v/v*) to obtain the desired (*S,S*)-product (**5**, 32% yield), the (*S,R*)-by-product (34% yield) and a mixture of diastereoisomer (13% yield). [The separation of the diastereomers can be also performed by semi-preparative TLC with SIL G-200 / UV_{254}, ethyl acetate/petrolether, 1/1, *v/v*]. ^{1}H-NMR (400 MHz, $CDCl_3$): δ = 8.90 (s, 1H), 8.66 – 8.54 (m, 2H), 8.49 (s, 1H), 7.94 (s, 1H), 7.42 (d, *J* = 8.0 Hz, 1H), 7.35 – 7.21 (m, 2H), 7.09 (td, *J* = 7.6, 1.3 Hz, 2H), 7.00 (s, 1H), 6.50 (s, 1H), 4.63 (s, 1H), 4.05 (t, *J* = 7.4 Hz, 1H), 2.80 (dh, *J* = 13.4, 6.1 Hz, 1H), 2.68 – 2.41 (m, 3H), 2.27 – 2.00 (m, 3H), 1.80 (s, 1H). ^{19}F-NMR (377 MHz, $CDCl_3$): δ = -82.07 – -89.48 (m), -96.84 – -97.89 (m). LC-MS (gradient mode A): purity = 98%, t_R = 10.9 min, m/z (ESI^+) = 642.91/644.91 (calcd. 643.07/ 645.06 for $[M+H]^+$ with $^{79}Br/^{81}Br$), m/z (ESI^-) = 640.87/642.84 (calcd. 641.05/643.05 for $[M-H]^-$ with $^{79}Br/^{81}Br$).

(*S*)-*N*-((*S*)-1-(2-chlorophenyl)-2-((3,3-difluorocyclobutyl)amino)-2-oxoethyl)-1-(4-cyanopyridin-2-yl)-5-oxo-*N*-(5-(tributylstannyl)pyridin-3-yl)pyrrolidine-2-carboxamide (**6**): The (*S,S*)-compound **5** (250 mg, 388 µmol 1 eq), bis(tributyltin) (Sn_2Bu_6, 215 µL, 427 µmol, 1.1 eq), palladium(II) acetate ($Pd(OAc)_2$, 0.9 mg, 3.88 µmol, 0.01 eq), tricyclohexylphosphine (PCy_3, 2.2 mg, 7.77 µmol, 0.02 eq) and 2 mL DMF were heated in a sealed tube at 110 °C for 24 h (TLC/SiO_2 in ethyl acetate/petrolether 1/1, *v/v*, R_f = 0.46). After the addition of water (10 mL), the mixture was extracted with ethyl acetate (3 x 10 mL), dried over anhydrous $MgSO_4$, filtered and evaporated to dryness. The crude product was purified by flash chromatography (silica, gradient ethyl acetate/petrolether 1/3 → 1/2 → 1/1 → 2/1 → 3/1, *v/v*) to give **6** as white solid (45%). ^{1}H-NMR (400 MHz, $CDCl_3$): δ = 8.93 (d, *J* = 2.4 Hz, 1H), 8.75 – 8.61 (m, 1H), 8.55 – 8.29 (m, 2H), 7.58 (s, 1H), 7.29 – 7.19 (m, 2H), 7.14 (t, *J* = 7.7 Hz, 1H), 7.03 – 6.81 (m, 2H), 6.40 (s, 1H), 6.19 (dd, *J* = 41.8, 6.8 Hz, 1H), 4.81 (ddd, *J* = 39.6, 9.3, 3.1 Hz, 1H), 4.33 (ddt, *J* = 8.3, 4.6, 1.5 Hz, 1H), 3.11 – 2.74 (m, 3H), 2.64 – 1.82 (m, 5H), 1.69 – 0.74 (m, 27H). ^{19}F-NMR (377 MHz, $CDCl_3$): δ = -84.70 (ddtd, *J* = 198.3, 41.6, 13.2, 6.5 Hz), -97.42 (ddq, *J* = 199.7, 145.8,

13.6 Hz). LC-MS (isocratic mode): purity = 99%, t_R = 15.0 min, m/z (ESI^+) = 855.35 (calcd. 855.2618 for $[M+H]^+$) and 896.31 (calcd. 896.23 for $[M+MeCN+H]^+$), m/z (ESI^-) = 853.31 (calcd. 853.25 for $[M+H]^+$). HRMS: m/z (ESI^+) = 855.2622 (calcd. 855.2618 for $[M+H]^+$).

Chemical synthesis of the diastereomeric mixture of (*S*,*R*)-AG-120 and (*S*,*S*)-AG-120

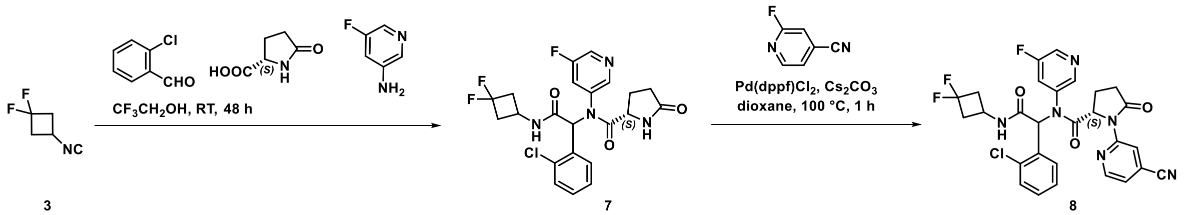

(2*S*)-*N*-(1-(2-chlorophenyl)-2-((3,3-difluorocyclobutyl)amino)-2-oxoethyl)-N-(5-fluoropyridin-3-yl)-5-oxopyrrolidine-2-carboxamide (**7**): A mixture of 2-chlorobenzaldehyde (60 mg, 0.4 mmol, 1 eq), 5-fluoropyridin-3-amine (40 mg, 0.4 mmol, 1 eq) in 2-fluoroethanol (0.5 mL) was stirred at RT for 30 min with molecular sieve. Then, (*S*)-5-oxopyrrolidine-2-carboxylic acid (84 mg, 0.4 mmol, 1 eq) was added and the mixture was stirred again at RT for 15 min. After addition of 1,1-difluoro-3-isocyanocyclobutane (**3**, 50 mg, 0.4 mmol, 1 eq), the reaction mixture was stirred at RT for 2 days. The mixture was concentrated under reduced pressure and the residue was purified by flash chromatography (RP-silica, gradient MeOH/H_2O 1/1→ 1.5/1 → 2/1 → 1/1, *v/v*) to afford the desired product **7** as yellow solid (87% yield, mixture of two diastereomers), which was used directly in the next step. LC-MS (gradient mode B): purity = 99%, t_R = 9.3 min, m/z (ESI^+) = 522.28 (calcd. 522.15 for $[M+MeCN+H]^+$), m/z (ESI^-) = 479.42 (calcd. 479.11 for $[M-H]^-$).

(2*S*)-*N*-(1-(2-chlorophenyl)-2-((3,3-difluorocyclobutyl)amino)-2-oxoethyl)-1-(4-cyanopyridin-2-yl)-N-(5-fluoropyridin-3-yl)-5-oxopyrrolidine-2-carboxamide (**8**): A solution of the diastereomeric mixture **7** (140 mg, 0.3 mmol, 1 eq), 2-fluoroisonicotinonitrile (64 mg, 0.3 mmol, 1.2 eq), caesium carbonate (101 mg, 0.3 mmol, 1.2 eq), 4,5-bis(diphenylphosphino)-9,9-dimethylxanthene (Xantphos, 8 mg, 0.01 mmol, 0.05 eq) and [1,1′-bis(diphenylphosphino)ferrocene]dichloropalladium(II) (9 mg, 0.01 mmol, 0.05 eq) in dioxane (2 mL) was stirred at 100 °C for 1 h. After concentration of the mixture under pressure, the residue was dissolved in ethyl acetate (50 mL), washed with water (3 x 20 mL) and brine (20 mL), dried over anhydrous $MgSO_4$, filtrated and concentrated under reduced pressure. Then, the raw product was purified by flash chromatography (silica, gradient ethyl acetate/PE, 1/2 → 1/1.5 → 1/1, *v/v*). The diasteromeric mixture of (*S,S*)-**7** and (*S,R)*-**7** was obtained as yellow solid (73% yield). ^{1}H-NMR (400 MHz, $CDCl_3$): δ = 8.75 – 8.63 (m, 2H), 8.63 – 8.44 (m, 2H), 8.42 – 8.03 (m, 4H), 7.45 – 7.24 (m, 6H), 7.24 – 6.83 (m, 6H), 6.64 – 5.93 (m, 4H), 4.92 – 4.57 (m, 2H), 4.40 – 4.00 (m, 2H), 3.10 – 1.93 (m, 16H). ^{19}F-NMR (282 MHz, $CDCl_3$): δ = -84.68 (d, *J* = 199.9 Hz), -97.67 (t, *J* = 201.9 Hz), -122.56 – -127.84 (m). LC-MS (gradient mode B): purity = 99%, t_R = 9.3 min, m/z (ESI^+) = 583.22 (calcd. 583.15 for $[M+H]^+$), m/z (ESI^-) = 581.37 (calcd. 581.13 for $[M-H]^-$).

Radiochemistry

General

No-carrier-added [^{18}F]fluoride was produced via the [^{18}O(p,n)^{18}F] nuclear reaction by irradiation of an [^{18}O]H_2O target (Hyox 18 enriched water, Rotem Industries Ltd, Israel) on a Cyclone 18/9 (iba RadioPharma Solutions, Belgium) with fixed energy proton beam using Nirta [^{18}F]fluoride XL target. Analytical chromatographic separations were performed on a JASCO LC-2000 system, incorporating a PU-2080Plus pump, AS-2055Plus auto injector (100 µL sample loop), and a UV-2070Plus detector (Jasco Deutschland GmbH, Pfungstadt, Germany) coupled with a gamma radioactivity HPLC detector (Gabi Star, Elysia-raytest GmbH, Straubenhardt, Germany). Data analysis was performed with the Galaxie chromatography software (Agilent Technologies).

Radiosynthesis of [^{18}F]FET

Results: The radiosynthesis of [^{18}F]**FET** was performed using a TRACERlab FX2 N synthesizer via a two step one pot procedure as already described [2,3]. In the first step, radiofluorination of the protected tosylate precursor TET was performed using the conventional [^{18}F]F^-/kryptofix222/carbonate system in acetonitrile followed by deprotection with 2 M HCl. Interestingly, only 1 mg of precursor was needed to achieve high radiochemical conversions (RCC) of 85±4 % (n=3) which is in contrast to the results of Bourdier et al. reporting on an optimized precursor amount of 6 mg [3]. For purification of the product, semi-preparative radio-HPLC was performed using a reversed phase column with 10% ethanol/50 mM aqueous phosphate (pH 6.5) as eluent (chromatogram in Figure S1A). In this way, [^{18}F]**FET** was produced in a total synthesis time of 60 min with a radiochemical purity of ≥99% (chromatogram in Figure S1B) and a radiochemical yield of 56 ± 7% (n=5, EOB). The molar activities were in the range of 30-80 GBq/µmol (calculated to the end of synthesis EOS) at starting activities of 4-13 GBq.

Experimental: For the radiosynthesis of [^{18}F]**FET** the protected tosylate precursor tert-butyl (S)-3-(4-(2-(tosyloxy)ethoxy)phenyl)-2-(tritylamino)propanoate (TET from ABX advanced biochemical compounds GmbH, Radeberg, Germany) was used. For identification of the radioactive product, (S)-2-amino-3-(4-(2-fluoroethoxy)phenyl)propanoic acid (FET from ABX advanced biochemical compounds GmbH, Radeberg, Germany) was used as reference compound. The radiosynthesis was performed using a TRACERlab FX2 N synthesizer (GE Healthcare, USA) equipped with a Laboport vacuum pump N810.3FT.18 (KNF Neuburger GmbH, Freiburg, Germany), a BlueShadow UV detector 10D (KNAUER GmbH, Berlin, Germany) and the TRACERlab FX Software.

[^{18}F]Fluoride (4 – 13 GBq) was trapped on a Sep-Pak® Accell QMA light cartridge (Waters GmbH, Eschborn, Germany) and eluted into the reactor with 1100 µL of a solution of Kryptofix 222 (K_{222}, 1.8 mg, 13 µmol), K_2CO_3 (11 mg, 29 µmol) in a mixture of 300 µL water and 800 µL MeCN. After azeotropic distillation for 5 min at 75 °C, 1.5 mL of MeCN was added and the mixture was further azeotropically dried for 2 min at 95 °C. Thereafter, the dried [^{18}F]fluoride-complex was cooled to 40 °C, 1.0 mg (1.5 µmol) of the precursor TET dissolved in 750 µL of anhydrous MeCN was added and the

reaction mixture was stirred at 100 ºC for 10 min. After cooling to 40 °C, 800 µL of an aqueous 2 M HCl solution was added and deprotection was performed within 7 min at 110 °C. After cooling to 30 °C, the reaction mixture was neutralized with 1.6 mL of an aqueous 1M $NaHCO_3$ solution and 1.8 mL of an aqueous 100 mM phosphate buffer (pH 6.5). After stirring for 2 min, the solution was subjected to semi-preparative radio-HPLC using a Reprosil-Pur 120 C18-AQ column (250 x 10 mm; 10 µm; Dr. Maisch HPLC GmbH, Germany) with a solvent composition of 10% ethanol/50 mM aqueous phosphate (pH 6.5) at a flow rate of 2.3 mL/min (Fig. S1A). About 5 mL of product fraction was collected in a 10 mL V-vial and transferred out of the hot cell. At a starting activity of 6 GBq, about 2 GBq of [^{18}F]**FET** could be produced in a total synthesis time of 60 min, with a radiochemical purity ≥ 99% and a radiochemical yield of 56 ± 7% (n=5, EOB). The molar activities were in the range of 30 - 80 GBq/µmol (calculated to the end of synthesis).

The identity of [^{18}F]**FET** has been confirmed by analytical radio-HPLC of the final product spiked with the reference compound **FET** (Fig. S1B). The molar activities were determined on the basis of a calibration curve carried out under isocratic HPLC conditions (10% ethanol/50 mM aqueous phosphate (pH 6.5) at a flow rate of 0.75 mL/min; Reprosil-Pur 120 C18-AQ, 250 x 4.6 mm) using chromatograms obtained at 224 nm as a wavelength with high absorption.

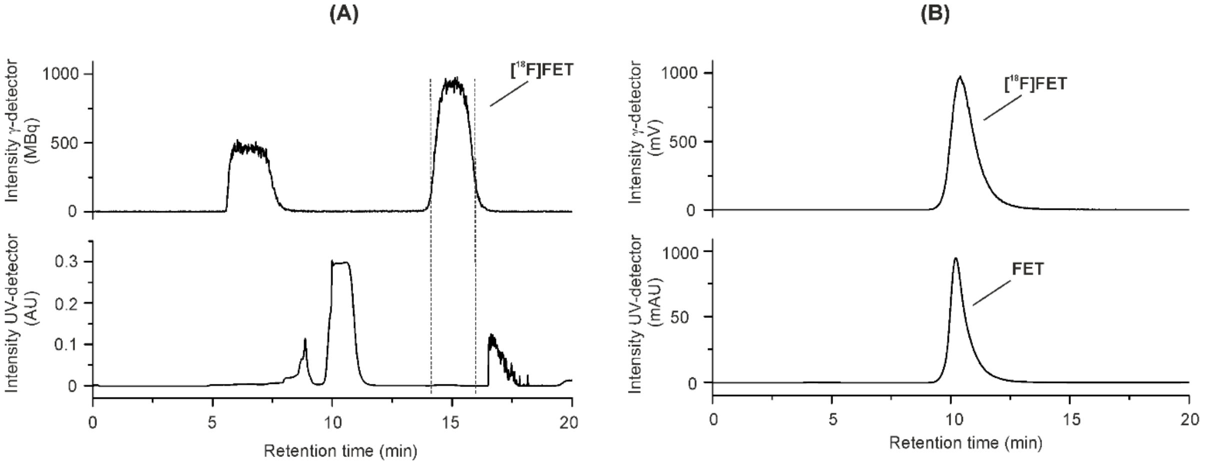

Fig. S1 (**A**) Exemplary radio- and UV-HPLC chromatograms of the semi-preparative HPLC purification of [^{18}F]**FET** (conditions: Reprosil-Pur 120 C18-AQ column (250 x 10 mm), 10% ethanol/50 mM aqueous phosphate (pH 6.5), 2.3 mL/min) (**B**) analytical radio- and UV-HPLC chromatograms of the final product spiked with non-radioactive FET (conditions: Reprosil-Pur 120 C18-AQ column (250 x 4.6 mm), 10% ethanol/50 mM aqueous phosphate (pH 6.5), 0.75 mL/min)

Radiosynthesis of [^{18}F]AG-120

General

The copper-mediated radiofluorination (CMRF) of the stannyl precursor **6** was manually investigated by using the fluorination agents *n*-tetrabutylammonium [^{18}F]fluoride ([^{18}F]TBAF), 4-(dimethylamino)pyridinium [^{18}F]fluoride ([^{18}F]DMAPHF) and sodium [^{18}F]fluoride ([^{18}F]NaF) [4–8]. As copper source tetrakis(pyridine)copper(II) bis(trifluoromethanesulfonate) ([Cu(OTf)$_2$(py)$_4$]) was used. Sodium trifluoromethanesulfonate (NaOTf), *n*-tetrabutylammonium trifluoromethanesulfonate (TBAOTf) and anhydrous methanol were purchased from VWR International GmbH, Part of Avantor, Darmstadt, Germany. The anhydrous labeling solvents 1,3-dimethyl-2-imidazolidinone (DMI) and *N*,*N*-dimethylacetamide (DMA) as well as 4-dimethylaminopyridine (DMAP), trifluoromethanesulfonic acid (HOTf) and [Cu(OTf)$_2$(py)$_4$] were purchased from Sigma-Aldrich Chemie GmbH, Part of Merck, Taufkirchen, Germany. The *n*-tetrabutylammonium hydrogencarbonate (TBAHCO$_3$) was used as 0.075 M solution from ABX advanced biochemical compounds GmbH, Radeberg, Germany. The salt 4-(dimethylamino)pyridinium trifluoromethanesulfonate (DMAPHOTf) was synthesized according to the procedure described by Zhang et. al using DMAP and HOTf [5].

Radio thin layer chromatography (radio-TLC) was performed on silica gel (Polygram® SIL G/UV$_{254}$ from Machery-Nagel, Germany) pre-coated plates with a mixture of ethyl acetate/*n*-hexane 2/1 (v/v) as eluent. The plates were exposed to storage phosphor screens (BAS-IP MS 2025, FUJIFILM Co., Tokyo, Japan) and recorded using the Amersham Typhoon RGB Biomolecular Imager (GE Healthcare Life Sciences). Images were quantified with the ImageQuant TL8.1 software (GE Healthcare Life Sciences).

For analytical HPLC a Reprosil-Pur 120 C18-AQ column (250 x 4.6 mm; 5 µm; Dr. Maisch HPLC GmbH; Germany) with MeCN/20 mM NH_4OAc aq. (pH 6.8) as eluent mixture and a flow of 1.0 mL/min was used (gradient: eluent A 10% MeCN/20 mM $NH_4OAc_{aq.}$; eluent B 90% MeCN/20 mM $NH_4OAc_{aq.}$; 0–5 min 100% A, 5–20 min up to 100% B, 20–24 min 100% B , 24–25 min up to 100% A, 25–30 min 100% A). The ammonium acetate concentration stated as 20 mM $NH_4OAc_{aq.}$ corresponds to the concentration in the aqueous component of an eluent mixture. For the determination of molar activities, an aliquot of the tracer solution (30 µL, 25 - 30 MBq) was analysed by HPLC under isocratic conditions (50% MeCN/20 mM $NH_4OAc_{aq.}$; Reprosil-Pur 120 C18-AQ, 250 x 4.6 mm) using chromatograms obtained at 244 nm as the wavelength of high absorption. The amount of non-radioactive AG-120 was calculated from a calibration curve obtained under the same HPLC conditions.

Manual radiofluorination experiments

Tab. S1 CMRF conditions and RCCs of the attempts to synthesize [^{18}F]**AG-120**

**Elution agent**	**Entry**	**Solvent**	**Temp. (°C)**	**6 (µmol)**	**Ratio 6 to [Cu(OTf)$_2$(py)$_4$]**	**RCC (%)** [a]
**Without azeotropic drying procedure**						
DMAPHOTf [b]	1	DMI	115	3.5	1 : 3	**0.7**
	2	DMI	115	5.8	1 : 2	**0.3**
TBAOTf [c]	3	DMA	120	3.5	1 : 3	**3**
	4	DMI	140	3.5	1 : 3	**10**
	5 [d]	DMI	140	3.5	1 : 3	**5**
	6	DMI	140	2.3	1 : 3	**3**
**With azeotropic drying procedure**						
TBAHCO$_3$ [e]	7	DMA	120	5.8	1 : 2	**3**
	8	DMA	140	3.5	1 : 3	**5**
	9	DMI	140	3.5	1 : 3	**6**
NaOTf / K$_2$CO$_3$ [f]	10	DMA	120	3.5	1 : 3	**4**

[a] RCCs of [^{18}F]**AG-120** were determined by radio-TLC of samples taken from the reaction mixture after 5 min,

[b] generally 37 µmol (5 mg) DMAPHOTf were used,

[c] generally 13 µmol (5 mg) TBAOTf were used,

[d] 26 µmol (10 mg) TBAOTf were used,

[e] 3.75 µmol (50 µL of an 0.075 M solution) TBAHCO$_3$ were used,

[f] 58 µmol (10 mg) NaOTf and 0.36 µmol (0.05 mg) K$_2$CO$_3$ were used.

Manual experiments without azeotropic drying

No carrier added [^{18}F]fluoride in 1.0 mL of water was trapped on a Chromafix® 30 PS-HCO$_3^-$ (45 mg) cartridge (ABX advanced biochemical compounds GmbH, Radeberg, Germany) which was preconditioned by rinsing with 5 mL of an aqueous 0.5 M solution of NaHCO$_3$ and 10 mL water. After loading, the cartridge was washed with 2.0 mL of anhydrous methanol and dried with a stream of nitrogen for 3 min. The activity was then eluted either with 5 mg (13 µmol) TBAOTf or 5 mg (37 µmol) DMAPHOTf (synthesized according to the procedure described by Zhang et. al [5]) dissolved in 500 - 600 µL of anhydrous methanol achieving activity recoveries of 80 - 90%. The methanol was evaporated under a stream of nitrogen at 60 °C [8,9]. The dried [^{18}F]TBAF or [^{18}F]DMAPHF was then dissolved in 400 µL of the respective solvent (DMA or DMI), treated with the copper catalyst [Cu(OTf)$_2$(py)$_4$] and stirred for 1 min at RT. The stannyl precursor **6** was dissolved in 400 µL of the appropriate solvent and added to the solution of copper catalyst and fluorination agent. The ^{18}F-labelings were performed at 115,

120 or 140 °C, respectively (entries 1 - 6 Tab. S1). For monitoring of the labeling progress, aliquots were taken at 5, 10 and 15 min and analyzed by radio-TLC and randomly by radio-HPLC.

Manual experiments with azeotropic drying

No carrier added [^{18}F]fluoride in 1.0 mL of water was trapped on a Chromafix 30 PS-HCO_3^- (45 mg) cartridge (ABX advanced biochemical compounds GmbH, Radeberg, Germany) which was preconditioned by rinsing with 10 mL of an aqueous 0.5 M solution of NaOTf and 10 mL water. For labeling reactions with the [^{18}F]NaF, the activity was eluted with 550 µL of an aqueous solution of sodium triflate (10 mg, 58 µmol) and potassium carbonate (0.05 mg, 0.36 µmol) according to Makaravage et al. into a 4 mL V-vial containing 1 mL of MeCN [7]. The aqueous [^{18}F]fluoride was dried by azeotropic distillation under vacuum and nitrogen flow within 7-10 min using a single mode microwave (75 W, at 50−60 °C, power cycling mode; Discover PETWave from CEM corporation, USA). Two aliquots of MeCN (2 x 1.0 mL) were added during the drying procedure and the final complex was obtained as a white solid. When [^{18}F]TBAF was generated, the aqueous [^{18}F]fluoride (200 - 400 µL) was directly added to 50 µL of an 0.075 M solution of $TBAHCO_3$ (ABX advanced biochemical compounds GmbH, Radeberg, Germany) dissolved in 1 mL MeCN and dried by azeotropic distillation as described above. The dried [^{18}F]NaF or [^{18}F]TBAF, respectively, was then dissolved in 400 µL of DMA for further labeling. Both fluorination agents were treated with the respective amount of [$Cu(OTf)_2(py)_4$] and stirred for 1 min at RT. The stannyl precursor **6** was dissolved in 400 µL DMA and added to the solution of copper catalyst and fluorination agent. The ^{18}F-labelings were performed at 120 or 140 °C, respectively (entries 7 - 10 Tab. S1). For monitoring of the labeling progress, aliquots were taken at 5, 10 and 15 min and analyzed by radio-TLC and randomly by radio-HPLC.

<u>Automated radiosynthesis of [^{18}F]**AG-120**</u>

Remote controlled radiosynthesis of [^{18}F]**AG-120** was performed using a TRACERlab FX2 N synthesizer (GE Healthcare, USA) equipped with a Laboport vacuum pump N810.3FT.18 (KNF Neuburger GmbH, Freiburg, Germany), a BlueShadow UV detector 10D (KNAUER GmbH, Berlin, Germany) and the TRACERlab FX Software.

[^{18}F]Fluoride (10 – 30 GBq) was trapped on a Chromafix 30 PS-HCO_3^- (45 mg) cartridge which was preconditioned by rinsing with 5 mL of an aqueous 0.5 M solution of $NaHCO_3$ and 10 mL water (Fig. S2, entry 1), washed with 2 mL anhydrous methanol (entry 3) into the [^{18}O]H_2O vial (entry 2) and dried for 3 min under vacuum and air stream via vial 1 (entry 3). Subsequently vial 1 (entry 3) was filled with TBAOTf (5.0 mg, 12.8 µmol) dissolved in 550 µL of anhydrous methanol via the septum cap using a syringe connected to a teflon tube with a canula. The [^{18}F]fluoride was eluted into the reactor and the methanol was evaporated at 60 °C under vacuum and argon stream for 2 min. Retaining the 60 °C, 7.1 mg (10.5 µmol) [$Cu(OTf)_2(py)_4$] dissolved in 400 µL DMI was added from vial 3 (entry 5) followed by the addition of 3.0 mg (3.5 µmol) **6** dissolved in 400 µL DMI from vial 2 (entry 4). The reaction mixture

was stirred at 140 °C for 4 min. After cooling to 30 °C, the reaction mixture was diluted with 10 mL water (entry 6) and passed through a Sep-Pak® C18 light cartridge (entry 9). The product was eluted with 1.5 mL MeCN (entry 8) into the reactor, dissolved with 3 mL of a mixture of H_2O/MeCN 2.5/0.5 (*v/v*, entry 7) and transferred into the injection vial (entry 10). Semi-preparative radio-HPLC was performed using a Reprosil-Pur 120 C18-AQ column (250 x 20 mm; 10 µm; Dr. Maisch HPLC GmbH, Germany) with a solvent composition of 60% MeCN/20 mM $NH_4OAc_{aq.}$ at a flow rate of 7.0 mL/min (entry 11, chromatogram Fig. S3). The fraction containing [^{18}F]**AG-120** was collected in the dilution vessel (entry 12) previously loaded with 40 mL of water. Solvent exchange was performed by passing the solution through a Sep-Pak® C18 light cartridge (entry 13), followed by washing with 2 mL of water (entry 14) and elution of [^{18}F]**AG-120** with 1.5 mL of EtOH (entry 15) into the product vial (entry 16). The ethanolic solution was transferred out of the hot cell and the solvent was reduced under a gentle argon stream at 70 ºC to a final volume of 10-50 µL. Afterwards the radiotracer was diluted in isotonic saline to obtain a final product containing 10% of EtOH (v/v, chromatogram in Fig. S4 A). The identity and isomeric purity of [^{18}F]**AG-120** was confirmed by analytical radio- and UV-HPLC in isocratic mode using the final product spiked with a mixture of the diastereomers (*S*,*S*)-**AG-120** and (S,*R*)-**AG-120** (Fig. S4 B). The separation of the two diastereomers could not be detected under the gradient mode conditions. Thus, both diastereomers were observed as a single peak (chromatogram not shown).

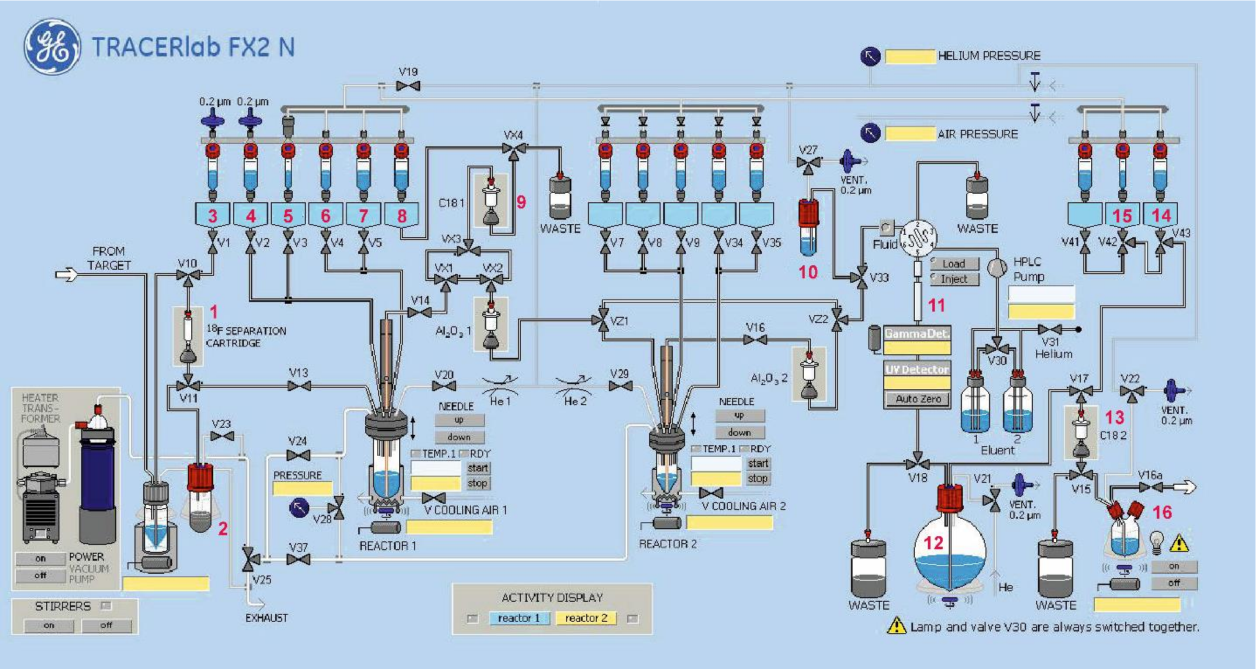

Fig. S2 Scheme of the synthesis module TRACERlab FX2 N for the radiosynthesis of [^{18}F]**AG-120**. (1) Chromafix 30 PS-HCO_3^-, (2) collection of [^{18}O]H_2O and methanol, (3) first 2 mL anhydrous methanol, second 5.0 mg TBAOTf in 550 µL anhydrous methanol, (4) 7.1 mg [Cu(OTf)$_2$(py)$_4$] in 400 µL DMI, (5) 3.0 mg **6** in 400 µL DMI, (6) 10 mL H_2O, (7) 3 mL H_2O/MeCN 2.5/0.5 (v/v), (8) 1.5 mL MeCN, (9) Sep-Pak® C18 light, (10) injection vial, (11) Reprosil-Pur 120 C18-AQ 250 × 20 mm (60% MeCN/20

mM $NH_4OAc_{aq.}$, flow 7.0 mL/min), (12) 40 mL water, (13) Sep-Pak® C18 light, (14) 2 mL water, (15) 1.5 mL EtOH, (16) product vial.

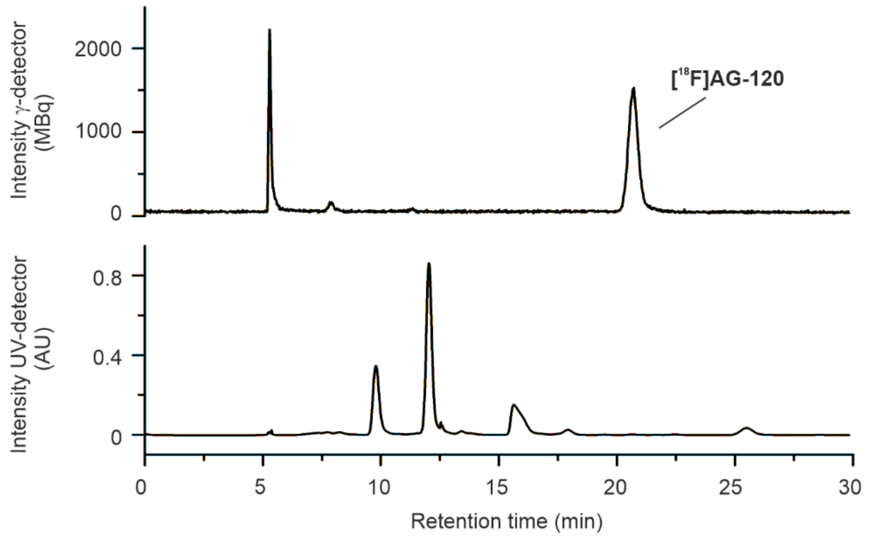

Fig. S3 Representative radio- and UV-chromatograms of the semi-preparative HPLC isolation of [^{18}F]**AG-120**.

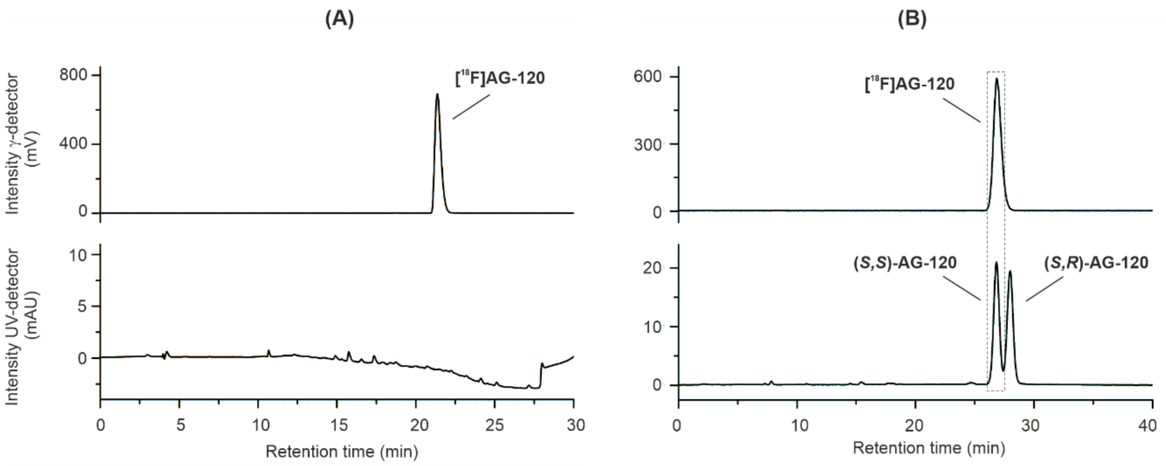

Fig. S4 Analytical radio- and UV-HPLC chromatograms of the formulated product [^{18}F]**AG-120** under gradient mode (**A**) and isocratic mode spiked with the diastereomeric mixture of (*S*,*S*)-**AG-120** and (*S*,*R*)-**AG-120** (**B**). Conditions: (A) Reprosil-Pur 120 C18-AQ (250 × 4.6 mm), MeCN/20 mM $NH_4OAc_{aq.}$, gradient mode with eluent A (10% MeCN/20 mM $NH_4OAc_{aq.}$) and eluent B (90% MeCN/20 mM $NH_4OAc_{aq.}$); 0–5 min 100% A, 5–20 min up to 100% B, 20–24 min 100% B , 24–25 min up to 100% A, 25–30 min 100% A, flow 1.0 mL/min; (B) Reprosil-Pur 120 C18-AQ (250 × 4.6 mm), isocratic mode with 46% MeCN/20 mM $NH_4OAc_{aq.}$, flow 1.0 mL/min.

Biological evaluation

General

Cyclosporine A (Sandimmune®), used to inhibit the P-glycoprotein (P-gp), was obtained from Novartis Pharma SAS, Rueil-Malmaison, France; **AG-120** was purchased from Axon Medchem, Groningen, The Netherlands and **BAY1436032** from Hucultec, Beutelbach, Germany.

Cell culture

Transfected human U-251MG glioblastoma cells stably overexpressing the human wildtype IDH1 (IDH1-U251) or the human IDH1R132H (IDH1R132H-U251), kindly provided by Dr. Jacqueline Kessler and Prof. Dirk Vordermark, Klinik für Strahlentherapie am Universitätsklinikum Halle Saale, Germany [10], were grown in RPMI 1640 medium supplemented with stable *L*-glutamine and 25 mM HEPES (Gibco™, #52400-025), 10% fetal bovine serum (Sigma-Aldrich, #F7524), 100 U/mL penicillin and 100 µg/mL streptomycin (Sigma-Aldrich, #P4333), and 5 µg/mL µg puromycin (Gibco™, #A11138-03)/mL in a humidified-air atmosphere incubator containing 5% CO_2 at 37 °C. Monolayer (2D) cultures were grown in flasks (25 cm^2; 175 cm^2) and 24-well plates depending on the experimental procedure.

Immunofluorescence

Immunocytofluorescence was performed 48 h after seeding at $2x10^5$/mL in an eight well micro-slide (Ibidi GmbH, Gräfeling, Germany). Cells were fixed in 2% PFA (paraformaldehyde) for 12 min at RT. Tissues were cryopreserved by incubation in 2-methylbutane at –25 °C (Merck, Germany). The brains were cut into coronal sections 8 µm thickness with cryostat (MICROM HM560, Fisher Scientific GmbH, Schwerte, Germany) and kept at –25 °C. Immunostaining was performed after fixation in PFA 4% for 20 min at 4 °C of the slides. Slides were washed twice with PBS and permeabilized for 10 min (0.3 % Triton X 100, in PBS). Following a 45 min blocking step (5% NGS, 0.2 % glycine, in PBST), the detection of the IDH1R132H or IDH1 was performed by 1 h RT incubation of the primary antibodies (DIAH09, 1/50 in blocking buffer; Biozol Diagnostica Vertrieb GmbH, Eching, Germany; and PA5-14358, 1/30 in blocking buffer, Invitrogen, Waltham, USA, respectively). After washing, the secondary antibodies (1/1000 in dilution buffer 1% BSA, Alexa Fluor Plus® 647 Goat-against-mouse #A32728 and Alexa Fluor Plus® 555 donkey-against-rabbit #A32794 respectively; Invitrogen, Waltham, USA) were incubated for 2 h at RT. A Hoechst counterstaining, 10 min at RT, was performed to visualize the nuclei of the cells (see above). After a step of washing and drying, slides were cover up with mounting medium (Aquapolymount, Polysciences Europe GmbH, Hirschberg an der Bergstrasse, Germany). Observation was performed by fluorescence microscopy (Leica, DMi8, software Leica LASX, Leica Mikrosysteme Vertrieb GmbH, Wetzlar, Germany) and image analysed by Image J [11].

Western Blotting

For Western blotting analyses, the IDH1-U251 and IDH1R132H-U251 cell extracts were prepared by lysing cells with RIPA buffer (#89900, ThermoScientific), supplemented with protease inhibitor (#87786, ThermoScientific) and phosphatase inhibitor (#78420, ThermoScientific), according to the manufacturers protocol. Lysates were separated by SDS-PAGE (10% ready-to-use gels; SERVA, #43264) and transferred to PVDF membranes by semi-dry blotting. The membranes were washed 2 x 5 min in TBS-T buffer (Roti®Stock 10x TBST C1061.1, Carl Roth GmbH, Karlsruhe, Germany), blocked for 1 h with 5% milkpowder (VT145.1, Carl Roth GmbH, Karlsruhe, Germany) in TBS-T (MP-TBS-T) and then incubated with the primary antibodies IDH1R132H (DIAH09, 1/250 in MP-TBS-T; Biozol Diagnostica Vertrieb GmbH, Eching, Germany) or IDH1 (PA5-14358, 1/250 in MP-TBS-T, Invitrogen, Waltham, USA) together with the GAPDH antibody (GTX89409, 1/1000 in MP-TBS-T, GeneTex, Irvine, CA, USA) overnight at 4 °C. The following day the membranes were washed 3 x in PBS-T, and incubated with the secondary antibodies (1/500 in TBS-T, Alexa Fluor Plus® 647 Goat-against-mouse #A32728 and Alexa Fluor Plus® 647 goat-against-rat #A45272 respectively; and Alexa Fluor Plus® 647 donkey-against-goat #A32849 Invitrogen, Waltham, USA) for 2 h at RT. After washing (3 x 5 min) the membranes were dried and the fluorescence signal was detected by a laser scanner (Amersham™Typhoon™ Amersham, UK).

IDH1 enzyme assay for determination of inhibitory potency

IDH1 enzyme assay is a modified version of the assay used for mIDH1. With the conversion of isocitrate to α-KG, this enzyme stoichiometrically converts NADP to NADPH. The produces NADPH which directly couples to the diaphorase/resazurin system and the resorufin production can be measured. The protocols for the determination of inhibition of IDH1 was done as described by Wang et al., (2013). The IDH1 (ab113858) was purchased from abcam (Cambridge, UK).

Briefly, IDH1 assays were conducted in 50 µL buffer (20 mM TRIS buffer (pH = 7.5), 150 mM NaCl, 10 mM $MgCl_2$, 0.05µµ% BSA and 4 mM β-mercaptoethanol) containing 50 µM NADP, 70 µM *DL*-isocitrate, and 0.04 µg/mL IDH1 enzyme (reaction time 1 h at RT). For inhibition assays, triplicate samples of the **AG-120** in the concentration range from 10^{-4} M to 10^{-10} M were incubated with the IDH1 for 1 h before addition of *DL*-isocitrate and NADP to initiate the reaction together with the direct detection system comprised of 20 µg/mL diaphorase and 4 µM resazurin. The reaction was terminated with by addition of 25 µL of 2% SDS and read on a Synergy H1 microplate reader at Ex544/Em590. The data were imported into GraphPad Prism and the IC_{50} values were calculated with a standard dose–response curve fitting (Fig. S6A).

Mutant IDH1 enzyme assay for determination of inhibitory potency

Determination of the activity and inhibition of mutant IDH1 recombinant protein, IDH1R132H, is based on the reduction of α-KG acid to D2HG accompanied by a concomitant oxidation of NADPH to NADP. The amount of NADPH remaining at the end of the reaction time is measured in a secondary

diaphorase/resazurin reaction in which the NADPH is consumed in a 1/1 molar ratio with the conversion of resazurin to the highly fluorescent resorufin. For the determination of inhibitory potential of ligands, the IDH1R132H Assay Kit (BPS-79376, BPS Bioscience, San Diego, CA, USA) was used. The enzyme activity assay was performed in a volume of 100 µl Buffer (20 mM TRIS buffer (pH = 7.5), 150 mM NaCl, 10 mM $MgCl_2$, 0.05% bovine serum albumin (BSA) and 4 mM *β*-mercaptoethanol) containing 0.5 ng/µL IDH1R132H enzyme, 2 mM α-KG, and 12 µM NADPH. For inhibition assays, triplicate samples of the AG-120 in the concentration range from 10^{-5} M to 10^{-10} M were incubated with the enzyme for 30 min prior to addition of α-KG and NADPH to initiate the reaction. The reaction run for 60 min at RT and was terminated with the addition of 25 µL of detection buffer (36 µg/mL diaphorase, 30 mM resazurin) to 50 µL of the reaction solution. The conversion of resazurin to resorufin by diaphorase measured fluorometrically at Ex544/Em590 (Synergy H1 microplate reader, BioTek. Winooski, VT, USA). The data were imported into GraphPad Prism 4.1 (GraphPad Inc.; La Jolla; CA), and the IC_{50} values were calculated with a standard dose–response curve fitting (Fig. S6B).

In vitro binding assays using [^{18}F]AG-120 and lysates of stably transfected U251 cells

The IDH1-U251 and IDH1R132H-U251cells were grown to confluency in a 175-cm^2 cell culture flask. The medium was removed and the cells were washed with 20 mL of PBS without Ca^{2+} and Mg^{2+} (Sigma-Aldrich; #RNBL1432). Cell detachment was performed by scraping the cells with fresh PBS. The cell suspension was transferred to a 50mL conical tube and centrifuged at 1200 × g for 3 min at RT. The supernatant was aspirated and discarded. The cell pellet was diluted with 500 µL of ice-cold hypotonic buffer (50 mM TRIS-HCl; pH 7.4 at 4 °C) and the suspension containing the lysed cells was stored at -28 °C until needed. On the day of the experiment, the required suspensions were thawed on ice, pooled, homogenized by passing twice through a 28-gauge needle, and diluted with the desired amount of incubation buffer (PBS without Ca^{2+} and Mg^{2+}, pH 7.4; Carl Roth, #9150.1; supplemented with 10 mM $MgCl_2$). The protein concentration of the final working solution was determined colorimetrically using a BCA assay (Pierce™, #23227). All binding experiments were performed by incubation of [^{18}F]**AG-120** with the cell lysates at a final concentration of ~ 360 µg protein/mL (corresponding to lysates of ~ 3.5 x 10^6 cells/mL) in 1 mL final volume at 220 rpm and RT (~ 21 °C) and were terminated by filtration through two layers of GF55 glass fiber filters (Hahnemühle, Germany) pre-wetted with deionized water followed by three washes with 1 mL cold 50 mM TRIS-HCl, pH 7.4 at 4 °C. Filter-bound radioactivity was measured by γ-counting (Wallac 2470 Wizard, Perkin Elmer, Waltham, MA, U.S.A). Assays were always performed in triplicate.

Association experiments [^{18}F]**AG-120** was incubated with IDH1R132H-U251 cell lysates without (total binding) and with 1 µM **BAY1436032** (non-specific binding) for various times up to 4 h.

Dissociation experiments [^{18}F]**AG-120** was incubated with IDH1R132H-U251 cell lysates without (total binding) and with 1 µM **BAY1436032** (non-specific binding) for 60 min. Dissociation was initiated by

addition of an excess of **AG-120** or **BAY1436032** (final concentratio n=0.9 µM) followed by incubation for various times up to 4 h.

Competition experiments [18F]**AG-120** was incubated with IDH1-U251 or IDH1R132H-U251 cell lysates with **AG-120** (homologous competition) or **BAY1436032** at increasing concentrations (10 pM–10 µM) for 60 min.

Data analyses were performed by non-linear regression using GraphPad Prism software to obtain association and dissociation rate constants and IC_{50} values. Apparent K_D and B_{max} values for [18F]**AG-120** were estimated by saturation curve analysis after correcting the measured data for the dilution of the molar activity of [18F]**AG-120** due to the addition of increasing concentrations of **AG-120**.

In vitro cell uptake studies using [18F]AG-120 and stably transfected U251 cells

The IDH1-U251 and IDH1R132H-U251cells were seeded at 400,000 cells/mL in a 24-well cell culture plate one day prior to the experiment. The medium was replaced by 400 µL/well and 4 µL of a 100-x stock solution of the respective inhibitor in 10% DMSO or vehicle (10% DMSO) were added about 2 h prior to the experiment. The experiment was started with the addition of 100 µL [18F]**AG-120** (6.1 ± 1.1 nM) diluted in cell culture medium per well, and the well plates were incubated in a humidified-air atmosphere incubator containing 5% CO_2 at 37 °C for various times. The incubation was stopped by aspiration of the supernatant and washing the cell layers twice with pre-chilled PBS (500 µL/well). Cell surface-bound activity was released by addition of acid-glycine buffer (0.2 M glycine, 0.15 M NaCl, pH 3; 500 µL/well) and incubation at RT for 10 min. The supernatant was collected and pooled with the supernatant obtained by subsequent washing with PBS (500 µL/well). Finally, the cells were lysed (0.1 M NaOH + 1% SDS; 500 µL/well; 37 °C, 30 min). Activities in the acidic wash and lysis samples along with aliquots of the radioligand solution were measured in a γ-counter (Wallac 2470 Wizard, Perkin Elmer, Waltham, MA, U.S.A). Cells cultured in an additional well plate and treated as above except for the addition of radioligand were used as a control and to determine the protein concentration per well by a BCA assay (Pierce™, #23227). The concentrations of surface-bound and internalized activities per well were calculated as a percentage of the applied dose per well and normalized to the protein concentration per well (% AD/µg protein). All experiments were performed in duplicates.

In vivo evaluation – General

Animal experiments were performed in accordance with the European guidelines for care of laboratory animals (Directive 2010/63/EU) and were approved by the Ethical Committee for Animal Testing at the University of Leipzig, Germany, and the regional authority Landesdirektion Sachsen, Germany (DD24.1-5131/446/19 and 25-5131/490/9). Female CD-1 mice (n=12, 10 weeks, 28-38 g) were obtained from the Medizinisch-Experimentelles Zentrum (MEZ) at Universität Leipzig (Leipzig, Germany) and male nude rats (n=6, 8 weeks old, 210 - 232 g) obtained from Janvier Labs (Le Genest-Saint-Isle, France).

In vivo metabolism in mice

Stability studies of [^{18}F]**AG-120** in female mice (8-10 weeks; 29-32 g; n=3) were performed as previously described [12]. The radiotracer was intravenously (i.v.) administered as bolus in awake CD-1 mice (27 ± 7 MBq [^{18}F]**AG-120)**. Brain and blood samples were obtained at 30 min post injection (p.i.), plasma was separated by centrifugation at 12,000 rpm at RT for 1 min (Centrifuge 5418; Eppendorf Vertrieb Deutschland GmbH; Wesseling-Berzdorf; Germany), and brain homogenized in 1 mL water on ice (10 strokes of a PTFE plunge at 1000 rpm in a borosilicate glass cylinder; Potter S Homogenizer; B. Braun Melsungen AG; Melsungen; Germany). All samples were weighed and the respective activity measured in a dose calibrator (ISOMED 2010; MED Nuklear-Medizintechnik Dresden GmbH; Dresden; Germany) [13].

RR-HPLC: For protein precipitation and extraction an ice-cold mixture of MeCN/water (4/1; *v/v*) was used in a ratio of 4/1 (*v/v*) of solvent to plasma or brain homogenate, respectively. The samples were vortexed for 3 min, equilibrated on ice for 5 min, and centrifuged for 5 min at 10,000 rpm. After separating the supernatant, the precipitates were washed with 100 µL of the solvent mixture and subjected to the same procedure. The combined supernatants were concentrated at 75 °C under nitrogen flow to a final volume of approximately 100 µL and analysed by analytical radio-HPLC. Separations were performed by using a Reprosil-Pur 120 C18-AQ column (250×4.6 mm; 5 µm; Dr. Maisch HPLC GmbH; Ammerbuch; Germany) and an eluent mixture of MeCN/20 mM $NH_4OAc_{aq.}$ (pH 6.8) in gradient mode (0–5' 10% MeCN, 5–20' up to 90% MeCN, 20–24' 90% MeCN, 24–25' up to 10% MeCN, 25–30 min 10% MeCN) at a flow rate of 1.0 mL/min. To determine the percentage of activity in the supernatants compared to total activity, aliquots of each step as well as the precipitates were quantified by a gamma counter (Wallac Wizard 1480; Perkin Elmer; Turku; Finland). With this procedure, recoveries ≥ 90% for plasma samples and brain homogenates could be obtained.

MLC: For preparation of the MLC samples, mouse plasma (20–50 µL) was dissolved in 200 µL of 200 mM aqueous sodium dodecyl sulfate (SDS) and injected into the MLC system. Separations were performed by using a Reprosil-Pur 120 C18-AQ column (250 x 4.6 mm + 10 mm pre-column, particle size: 10 µm) and an eluent mixture of 1-propanol/100 mM $SDS_{aq.}$/25 mM $(NH_4)_2HPO_4$ in gradient mode with eluent A: 100% 100 mM $SDS_{aq.}$/ 25 mM $(NH_4)_2HPO_{4,aq.}$ and eluent B: 30% 1-propanol/100 mM $SDS_{aq.}$/ 25 mM $(NH_4)_2HPO_{4,aq.}$ 0–10 min 100% A, 10–20 min up to 100% B, 20–30 min 100% B , 30–31 min up to 100% A, 31–40 min 100% A, flow 1.0 mL/min). The MLC system was built up of a JASCO PU-980 pump, an AS-2055Plus auto injector with a 2 mL sample loop, and a UV-1575 detector coupled with a gamma radioactivity HPLC detector (Gabi Star, raytest Isotopenmessgeräte GmbH; Straubenhardt; Germany). Data analysis was performed with the Galaxie chromatography software (Agilent Technologies).

Ex vivo P-glycoprotein efflux transporter study

For the time of the experiments, female CD-1 mice (n=10, 10 weeks, 28-38 g) were kept in a dedicated climatic chamber with free access to water and food under a 12:12h dark:light cycle at a constant

temperature (24 °C). intravenous (i.v.) injection were performed as bolus on awake animals. Permeability-glycoprotein efflux transporter studies consisted in pre-treatment via i.v. injection of cyclosporin A at 50 mg/kg (n=5) (Sandimmun®, Novartis Pharma SAS, Rueil-Malmaison, France) or of vehicle (NaCl/DMSO/kolliphor, 7/1/2, *v/v/v*; n=5) 30 min prior to [^{18}F]**AG-120** (0.40 ± 0.13 MBq in 150 µL isotonic saline; 0.2 ± 0.1 nmol/kg; A_m: 105-156 GBq/µmol, EOS, n=2). 5 min post-radiotracer injection, animals were euthanized, and blood and brain samples isolated. Plasma was separated by centrifugation at 12,000 rpm at RT for 1 min (Centrifuge 5418; Eppendorf Vertrieb Deutschland GmbH; Wesseling-Berzdorf; Germany). The activity in the different samples was measured in an automatic γ-counter (Wallac 2470 Wizard, Perkin Elmer, Waltham, MA, U.S.A). The % ID/g in the plasma, brain and blood were then calculated and brain-to-plasma ratio build.

Dynamic PET studies in naïve mice

For the time of the experiments, CD-1 mice (n=9, 8-10 weeks, 31-42 g) were kept in a dedicated climatic chamber with free access to water and food under a 12/12 h dark/light cycle at a constant temperature (24 °C). The animals were anaesthetized (anaesthesia unit U-410; agntho's; Lidingö; Sweden) with isoflurane (1.8%, 0.35 L/min) delivered in a 60% oxygen / 40% air mixture (Gas Blender 100 Series; MCQ instruments; Rome; Italy) and maintained at 37 °C with a thermal bed system. The P-gp efflux transporter studies consisted in pre-treatment via i.v. injection in the tail vein of cyclosporine A (Sandimmune®, 50 mg/kg, n=5) or of vehicle (NaCl/3% EtOH /6.6% kolliphor; n=4) 30 min prior to [^{18}F]**AG-120** (5.4 ± 0.7 MBq; 3.4 ± 2.3 nmol/kg; Am: 81-125 GBq/µmol, EOS, n=3), followed by a 60 min PET/MR scan (PET/MR 1Tesla; nanoScan®; MEDISO Medical Imaging Systems; Budapest; Hungary). Each PET image was corrected for random coincidences, dead time, scatter and attenuation (AC), based on a whole body (WB) MR scan. The list mode data were sorted into sonograms using a framing scheme of 12x10 s, 6x30 s, 5x300 s, 9x600 s. The reconstruction parameters for the list mode data were 3D-ordered subset expectation maximization (OSEM), 4 iterations, 6 subsets, energy window: 400-600 keV, coincidence mode: 1-5, ring difference: 81. Thereafter a T1 weighted WB gradient echo sequence (TR/TE: 20/6.4 ms, NEX: 1, FA: 25, FOV: 64x64 mm, matrix: 128x128, slice thickness: 0.5 mm) was performed for AC and anatomical orientation. Image registration and evaluation of the volume of interest (VOI) was done with PMOD v3.9 (PMOD technologies LLC; Zurich; Switzerland). The small intestine was delineated based on the PET image, and liver, heart, spleen, kidney and muscle were manually delineated based on the T1-weighted MRI inside the boundary of the organ to avoid spill-over effect. The activity data are expressed as mean standardized uptake value (SUV) of the overall ROI. Data are presented as mean ± standard deviation (SD).

Orthotopic glioma model

During the time of the experiment, animals were kept in a dedicated climatic chamber with free access to water and food under a 12/12 h dark/light cycle at a constant temperature of 24 °C. Four male nude rats (Rj:ATHYM-*Foxn1*$^{rnu/rnu}$; Janvier Labs, Le Genest-Saint-Isle, France) were used for tumor implantation at age of 8 weeks (210-232 g). During microsurgery the rats were anesthetized with a

mixture of air and isoflurane concentrate (1.5-2% depending on the breathing, anesthesia unit U-410; agntho's; Lidingö; Sweden) under aseptic conditions. A local anaesthesia of the scalp was performed by subcutaneous (s.c.) injection of 1% lidocaine (B. Braun, Melsungen, Germany) as well as a painkiller administration (Meloxicam, 1 mg/kg, s.c.) (Metacam®, Boehringer Ingelheim, Ingelheim am Rhein, Germany). The animals head was fixed into a stereotactic frame (Motorized New Standard Stereotaxic®, Stoelting, Dublin, Ireland). A midline incision was done and a burr hole was drilled 0.5 mm anterior and 2.7 mm lateral to the bregma. $5x10^6$ IDH1R132H-U251 or $1x10^6$ IDH1-U251 cells were suspended in 5 µL Hank's Buffer Salt Solution (HBSS 1X; Thermo scientific, Schwerte, Germany) and were injected 4.4 mm into the brain parenchyma with a flow rate of 0.5 µL/min using a 10 µL Hamilton syringe. After injection, the burr hole was filled with bonewax (Ethicon, US, LLC), the scalp incision sutured (Vicryl 6.0, Ethicon, US, LLC) and the surface antiseptically cleaned.

Next-Generation Sequencing

The tumor were resected 35 days post implantation and the fresh frozen tumor tissues underwent sequencing using the NGS protocol at University Hospital Dresden [14]. The tumor DNA was purified using the AllPrep® DNA Universal Kit for fresh frozen tissue (Qiagen, Germantown MD), following the manufacturer's instructions. The regions of interest were amplified using a custom-designed amplikon panel, according to the "QIAseq Targeted DNA V3 Panel, May 2017" protocol (QIAGEN, Hilden, Germany), which was custom-designed and manufactured by our group. The panel covered mutation hotspots or whole genes where loss of function is a known mechanism of action. The included glioma-relevant genes were *ATRX*, *BRAF*, *CDKN2A*, *DAXX*, *EGFR*, *IDH1*, *IDH2*, *NF1*, *PIK3CA*, *PIK3R1*, *PTEN*, *STAG2*, *TP53*, and *TERT* promotor. During library preparation, unique molecular barcodes and sample-specific indices were incorporated following the protocol. The indexed libraries were then quantified using a Qubit dsDNA HS Assay Kit (Thermo Fisher Scientific, MA, USA) and sequenced in paired-end mode (2x150 bp) on the Illumina NextSeq platform. HG19 was used as the reference genome for bioinformatic analyses. For all samples, further bioinformatics analysis was performed using the Biomedical Workbench from CLC (version 21.0.3) with a customized analysis algorithm. The analysis included filters such as coverage ≥100 and allele frequency ≥5%.

MRI monitoring

The tumor growth was monitored on a 7 T small animal imaging system BioSpec 70/30 (Bruker, Ettlingen, Germany) using a receive-only rat brain surface coil. T2-weighted image series were acquired using a TURBO-RARE sequence (TR/TE: 2212/33 ms; NEX: 6; FA: 90; FOV: 3.5 x 3 cm; matrix: 440 x 376; SI: 0.6 mm) in coronal plane to vizualize the tumor. Blood-brain barrier permeation was evaluated by two T1 weighted imaging using a FLASH sequence in the coronal plane (FLASH; TR/TE: 160/3 ms; NEX: 6; FA: 50; FOV: 4 x 4 cm; matrix: 384x384; SI: 0.8 mm) performed before and after the injection of a contrast agent at 0.2 mmol/kg i.v. (Gadovist®, Bayer, Leverkusen, Germany).

Dynamic PET studies in orthotopic brain tumor model

For the time of the experiments, the nude rats (n=4) were kept in a dedicated climatic chamber with free access to water and food under a 12/12 h dark/light cycle at a constant temperature (24 °C). The animals were anaesthetized with desflurane delivered in a 30% oxygen / 70% air mixture and maintained at 37 °C with a thermal bed system. [^{18}F]**AG-120** (38.7 ± 1.5 MBq in 200 µL isotonic saline; A_m: 223 GBq/µmol, EOS; 1.5 ± 0.1 nmol/kg) or [^{18}F]**FET** (36.6 ± 1.0 MBq in 200 µL isotonic saline; A_m: 79 GBq/µmol, EOS; 12.0 ± 0.3 nmol/kg) were injected into the tail vein followed by a 60 min PET/CT scan (NanoScan® PET/CT; MEDISO Medical Imaging Systems; Budapest; Hungary) at 30 and 34 days post-graft respectively. Each PET image was corrected for random coincidences, dead time, scatter and attenuation (AC), based on the CT scan. The list mode data were sorted into sonograms using a framing scheme of 15x10 s, 5x30 s, 5x60 s, 4x300 s, 3x600 s. The reconstruction parameters for the list mode data were TeraTomo 3D PET, 4 iterations, 6 subsets, energy window: 400-600 keV, coincidence mode: 1-5, ring difference: 81. Following the PET scan a T2 weighted scan was performed as described above. Image registration and evaluation of the volume of interest (VOI) was done with PMOD v3.9 (PMOD technologies LLC; Zurich; Switzerland). The blood compartment was delineated based on the CT showing the heart. The background signal was defined as a 1.5 mm sphere placed in the contralateral (left striatum). Region-specific activity uptake was assessed using dynamic and static parameters derived from the time-activity curves (TAC). Tumor-to-background ratios were calculated for the 30–60 min time frame ($TBR_{mean}=SUV_{mean(tumor)}/SUV_{mean(contralateral)}$).

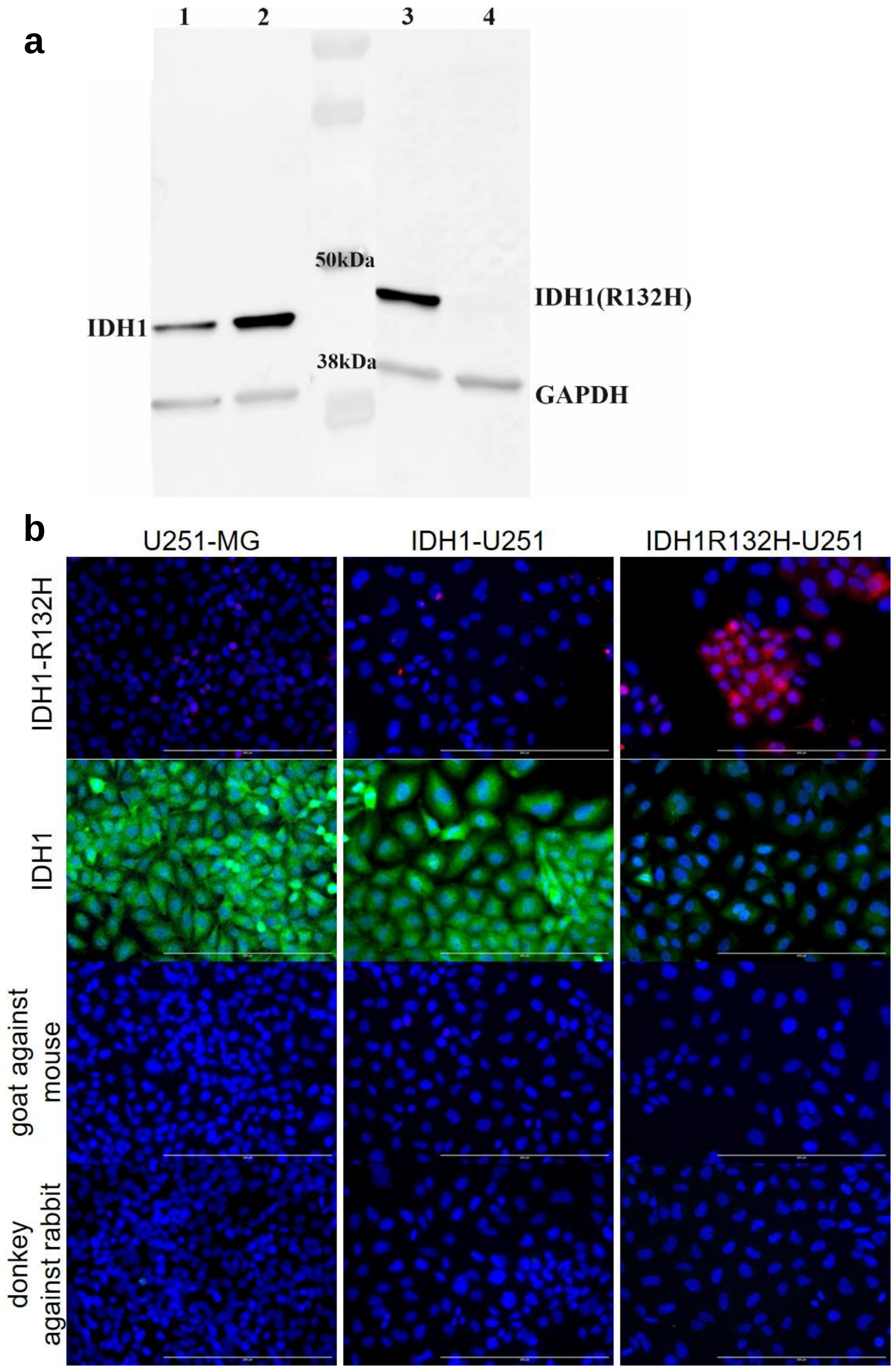

Fig. S5 Validation of the IDH1- and IDH1R132H U251 cells. IDH1 and IDH1R132H protein expression (**a**) in IDH1-U251 (lane 2-4) and IDH1R132H-U251 (lane 1-3). Western blot performed for detection of IDH1 (lane 1-2) and for IDH1R132H (lane 3-4). GAPDH served as loading control. Immunofluorescent microscopy (**b**). Representative merged images of the IDH1R132H staining (Dianova, DIA-H09) and of the IDH1 staining (Invitrogen, PA5-14358) in U251-MG, IDH1-U251 and IDH1R132H-U251 cells. Secondary antibodies controls are displayed. Scale bar: 200 μm, magnification x40; blue channel: nuclei staining merged with the green channel: IDH1 staining or the red channel: IDH1R132H staining.

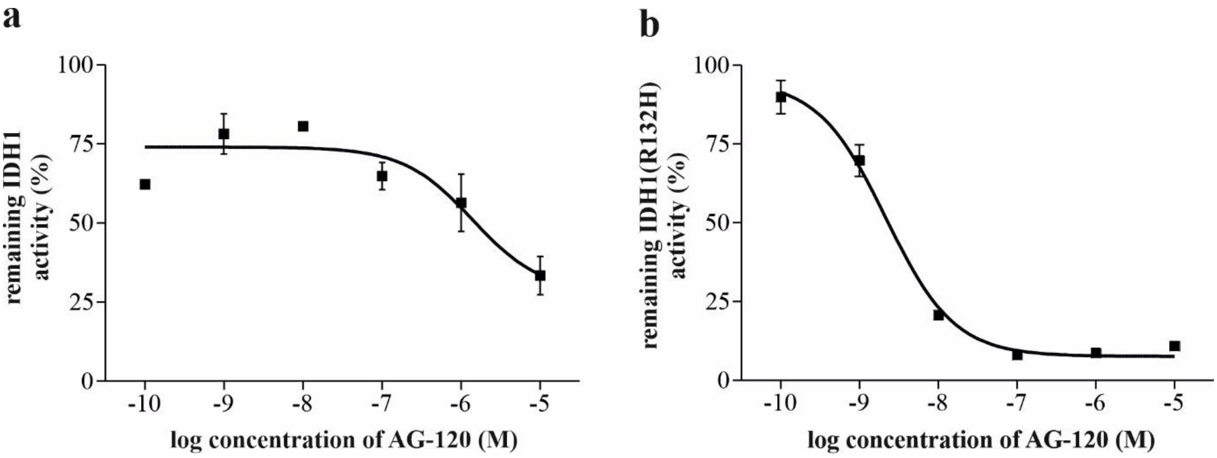

Fig. S6 Representative **AG-120** inhibition curves for (**A**) IDH1 and (**B**) IDH1R123H. Recombinant IDH1 and IDH1(R132H) proteins were preincubated with different concentrations (10^{-5} to 10^{-10}) of **AG-120**. One hour after the addition of the substrate the conversion of resazurin to resorufin by diaphorase was measured fluorometrically at Ex544/Em590. The IC_{50} values were calculated with a standard dose–response curve fitting: All experiments are performed in triplicates (n=3).

The inhibitory potential of **AG-120** for the IDH1 and IDH1R123H recombinant enzymes was evaluated. The IC_{50} values obtained were 0.147-6.85 µM and 4.35 ± 3.22 nM for IDH1 and IDH1R132H, respectively, in concordance with literature reports [1].

Tab. S2 Buffer composition of the in vitro binding assays

**Buffer name**	**Buffer composition**				
**A**	20 mM TRIS-HCl, pH 7.4	150 mM NaCl	10 mM $MgCl_2$		
**B**	20 mM TRIS-HCl, pH 7.4	150 mM NaCl	10 mM $MgCl_2$	0.05% BSA	
**C**	20 mM TRIS-HCl, pH 7.4	150 mM NaCl	10 mM $MgCl_2$	0.05% BSA	4 mM β-mercaptoethanol*
**D**	PBS, pH 7.4		10 mM $MgCl_2$		

* β-mercaptoethanol: prevent disulfide-formation

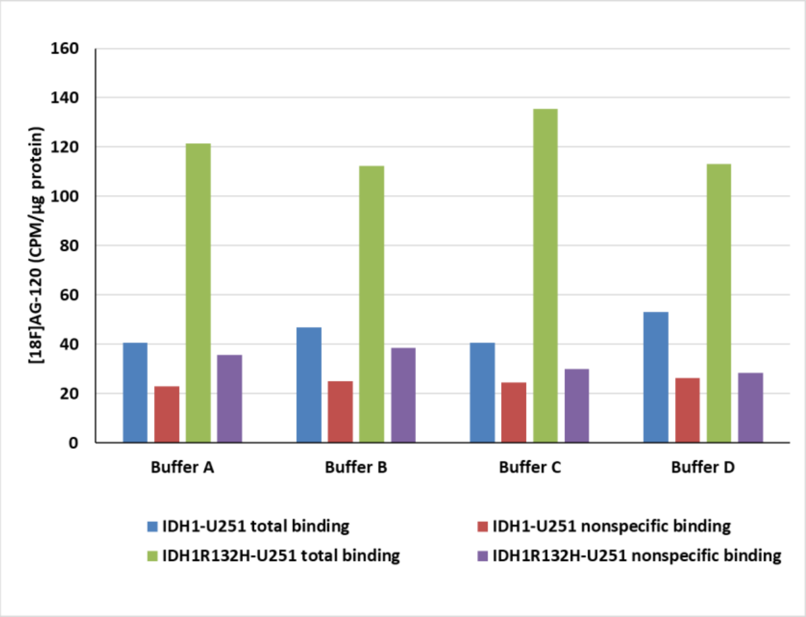

Fig S7 Comparison of the buffer composition on the [^{18}F]**AG-120** binding to IDH1-U251 and IDH1R132H-U251 cell lysates. Whole cell lysates of IDH1-U251 (0.275 mg protein/mL) and of IDH1R132-U251 (0.212 mg protein/mL) cells were incubated with 2.66 nM [^{18}F]**AG-120** without (total binding; triplicate) or with co-incubation with 1 µM **AG-120** (nonspecific binding; duplicate) at RT for 60 min. Results are presented as CPM/µg protein vs. the four different buffers (see Table S2).

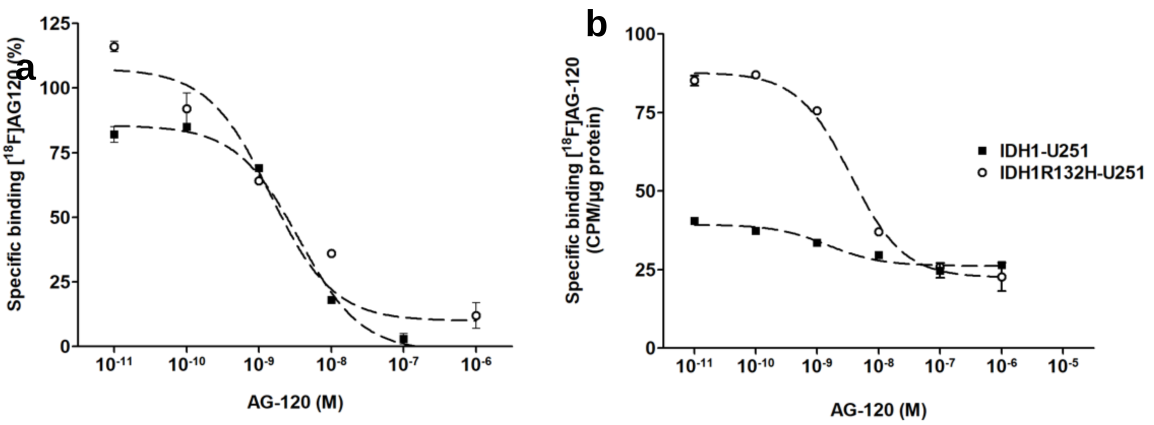

Fig. S8 Displacement of [^{18}F]**AG-120** binding to IDH1-U251 and IDH1R132H-U251 cell lysates by **AG-120**. Whole cell lysates of IDH1-U251 (0.448 mg protein/mL) and of IDH1R132-U251 (0.524 mg protein/mL) cells were incubated with 0.47 nM [^{18}F]**AG-120** for 60 min. Non-specific binding was determined by co-incubation with 1 µM **BAY1436032**. Results are presented as % of specific binding of the radioligand vs. concentration (**a**) and as concentration of specifically bound radioligand per µg protein vs. concentration of competitor (**b**). Displacement curves are fitted to a one-site model with estimated IC_{50} values of 1.51 nM and 3.52 nM (**a**), and estimated apparent apparent B_{max} values of 0.196 pmol/mg protein and 0.983 pmol/mg protein (**b**) for IDH1-U251 and IDH1R132H-U251 cell lysates, respectively.

Tab. S3 Tissue biodistribution of radioactivity at different time point after i.v. injection of [^{18}F]**AG-120** in CD-1 mice based on PET imaging (n=4).

	**Uptake (SUVmean)**				
	**5 min**	**15 min**	**30 min**	**45 min**	**60 min**
**blood**	0.90±0.21	0.73±0.12	0.57±0.06	0.49±0.05	0.44±0.11
**heart**	0.87±0.15	0.79±0.11	0.62±0.08	0.59±0.05	0.49±0.07
**lung**	0.48±0.09	0.47±0.09	0.40±0.06	0.36±0.05	0.33±0.03
**liver**	4.92±0.94	4.03±0.68	3.39±0.63	2.91±0.65	2.59±0.55
**gallbladder**	3.37±0.99	3.50±0.64	4.07±1.70	5.57±3.31	6.10±4.20
**stomach**	0.57±0.40	0.44±0.24	0.37±0.16	0.35±0.17	0.37±0.24
**small intestine**	1.76±0.45	2.00±0.52	2.28±0.89	2.70±1.15	2.85±1.21
**bladder**	0.23±0.20	0.86±0.76	1.68±1.37	1.99±1.31	1.99±1.10
**kidney**	1.69±0.26	1.37±0.11	1.16±0.10	1.11±0.05	1.07±0.08
**spleen**	1.03±0.28	0.90±0.14	0.73±0.09	0.62±0.16	0.57±0.11
**muscle**	0.45±0.18	0.54±0.10	0.51±0.05	0.44±0.04	0.40±0.05
**bones**	0.47±0.21	0.51±0.15	0.43±0.05	0.36±0.02	0.33±0.05
**brain**	0.07±0.02	0.11±0.02	0.11±0.02	0.11±0.02	0.10±0.02

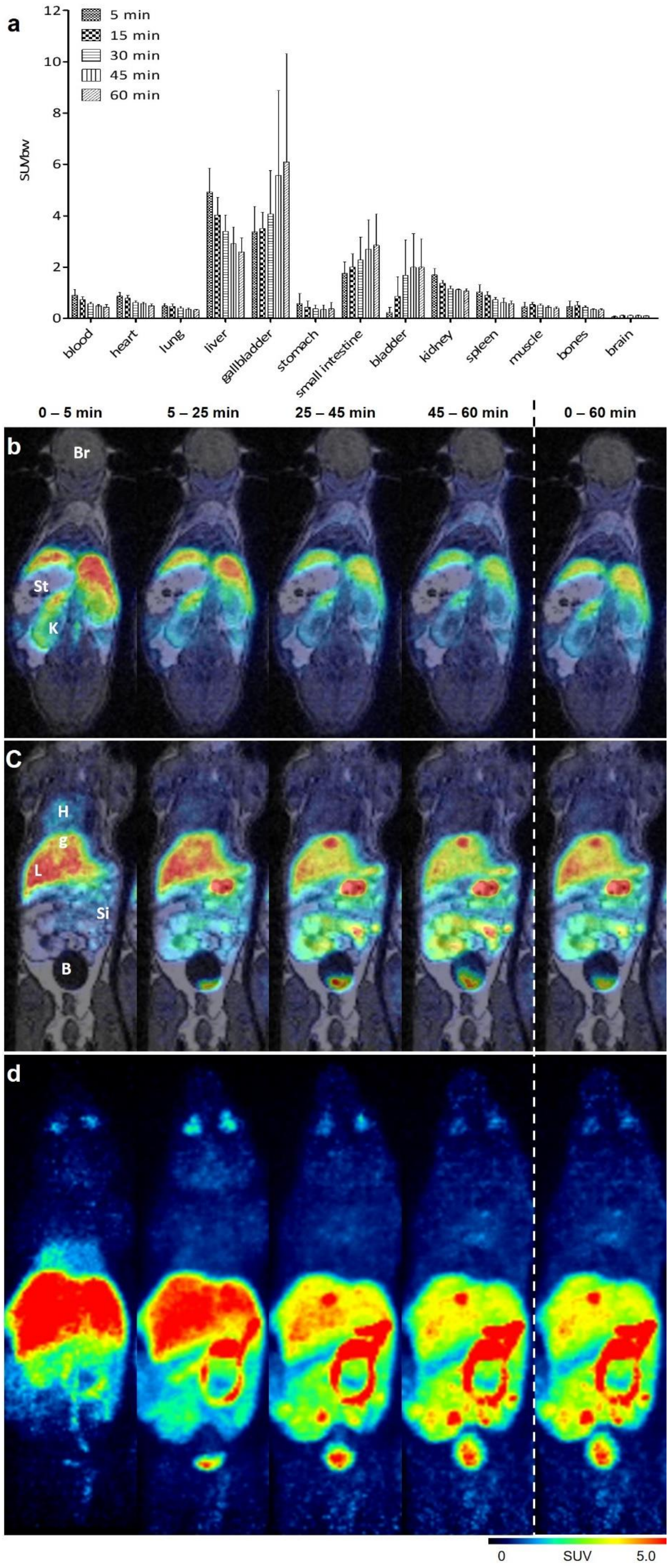
a
SUVbw
5 min
15 min
30 min
45 min
60 min
blood
heart
lung
liver
gallbladder
stomach
small intestine
bladder
kidney
spleen
muscle
bones
brain
0 – 5 min
5 – 25 min
25 – 45 min
45 – 60 min
0 – 60 min
b
Br
St
K
C
H
g
L
Si
B
d
0
SUV
5.0

Fig. S9 Biodistribution of [^{18}F]**AG-120** in CD-1 mice at different time points based on PET imaging (n=4) (**a**). Representative horizontal merged PET/MRI images of [^{18}F]**AG-120** biodistribution focused on (**b**) kidneys and stomach and on (**c**) liver, small intestine and bladder, and (**d**) corresponding maximal intensity projection (MIP). B: bladder; Br: brain; g: gallbladder; H: heart; K: kidneys; L: liver; Si: small intestine; St: stomach.

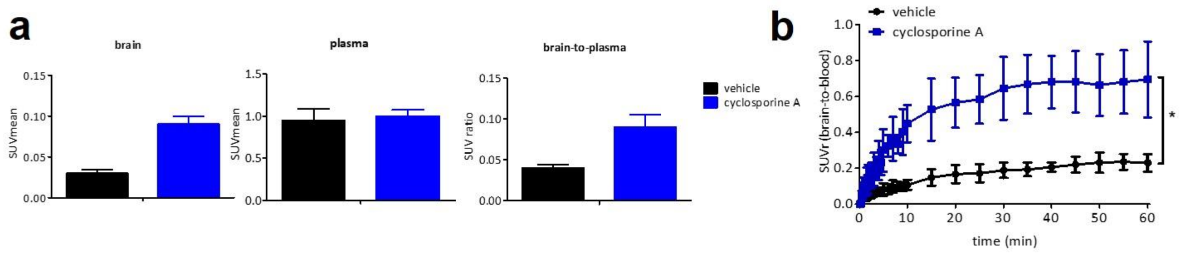

Fig. S10 Efflux transporter substrate study in naive CD-1 mice. (**a**) Ex vivo brain and plasma uptake (SUV_{mean}) 5 min post-administration of [^{18}F]**AG-120** in vehicle (black bars; n=5) and cyclosporine A (blue bars; n=4) pre-treated groups. (**b**) Tine-Activity Curves (TACs) of the SUV ratio (SUVr) brain-to-blood after pre-treatment with vehicle (black dot; n=4), or sandimmune® (50 mg/kg of cyclosporine A; blue square; n=5). Student T-test: * = p<0.001.

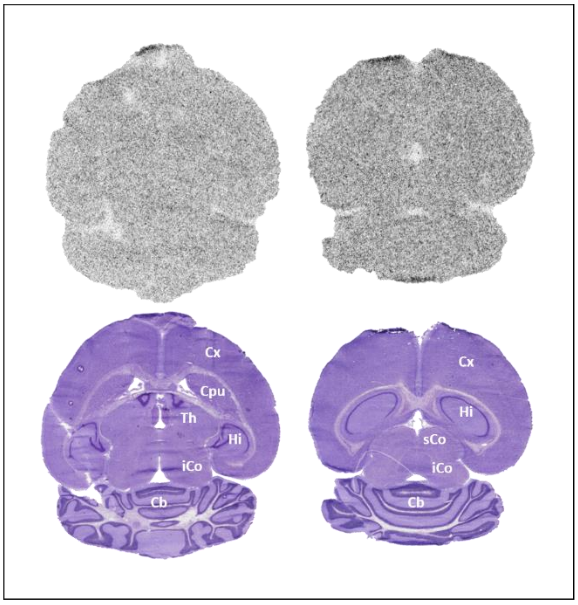

Fig. S11 Ex vivo autoradiography of one CD-1 mouse brain 70 min post [^{18}F]**AG-120** injection pre-treated with cyclosporine A (Sandimmune®, 50 mg/kg).

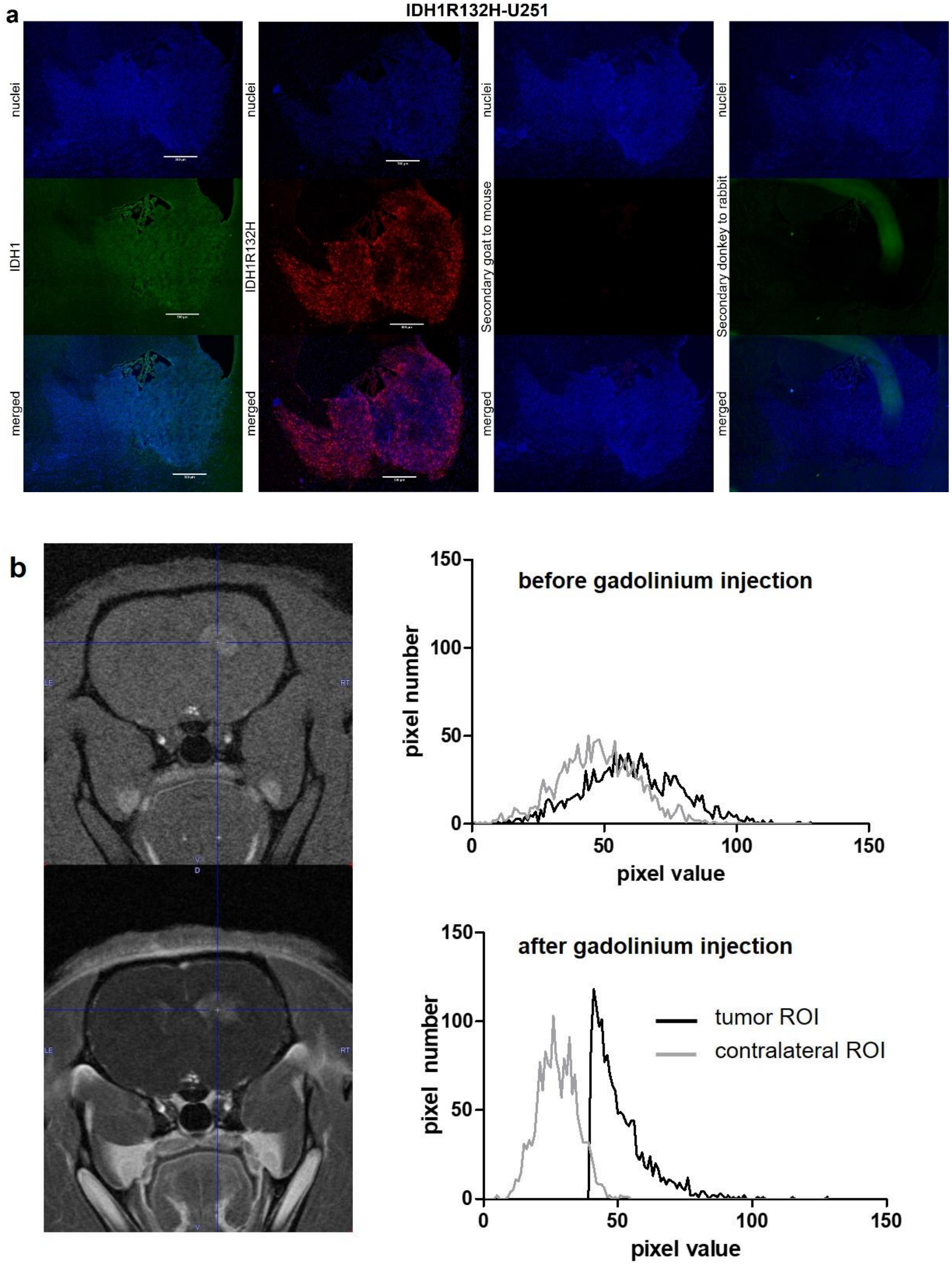

Fig. S12 Brain tumor characterization. (**a**) Immunofluorescent microscopy. Representative merged images of the IDH1R132H staining (Dianova, DIA-H09) and of the IDH1 staining (Invitrogen, PA5-14358) in IDH1R132H-U251 cells. Secondary antibodies controls are displayed. Scale bar: 500 µm, magnification x10; blue channel: nuclei staining merged with the green channel: IDH1 staining or the

red channel: IDH1R132H staining. (**b**) Exemplary T1-weighted images of a IDHR132H tumor before and after contrast agent injection (Gadovist® intravenously injected at 0.2 mmol/kg) and corresponding histogram of the tumor and contralateral region. The contralateral region is used as reference region. A shift in the pixel value distribution (i.e. pixel intensity) to the right corresponds to a contrast enhancement.

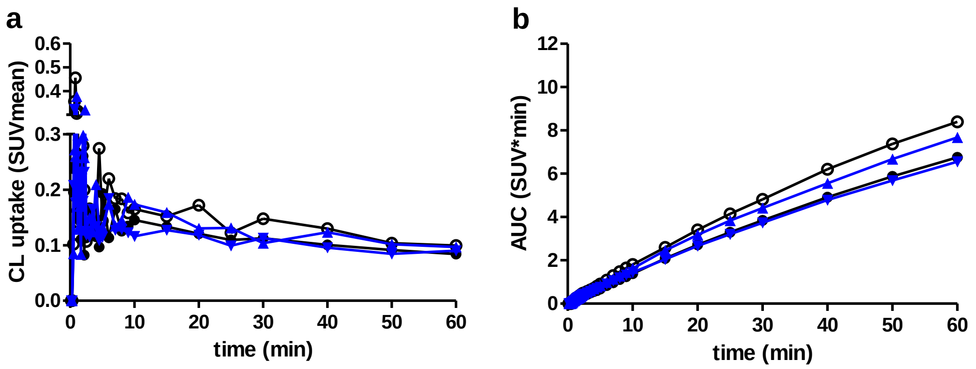

Fig. S13 TACs of [^{18}F]**AG-120** accumulation in SUV_{mean} (**a**) and AUC in SUV*min (**b**) of the contralateral (CL) of IDH1-tumor animals (black line, n=2) and IDH1R132H-tumor animal (blue line, n=2)

Tab. S4 Static and dynamic parameters derived from [^{18}F]**FET**-PET acquisition

[^{18}F]**FET**	parameter	R1-IDH1-WT	R2-IDH1-WT	**Mean IDH1-WT**	R3-IDH1-R132H	R4-IDH1-R132H	**Mean IDH1-R132H**
dynamic parameter (SUV_{mean} TACs)	Time-to-peak (min)	60	60	**60**	40	60	**50**
	TAC peak value (SUV)	0.85	0.89	**0.87**	0.99	0.85	**0.92**
	AUC_{60min} (SUV*min)	37.85	44.98	**41.42**	49.12	39.84	**44.48**
	r^2	0.82	0.30	**-**	0.57	0.90	**-**
	$slope_{30-60\ min}$ (SUV/min)	0.0054	0.0014	**0.0034**	-0.0024	0.0040	**0.0008**
Static parameter (30-60 min)	SUV_{peak}	0.81	0.97	**0.89**	0.98	0.77	**0.88**
	SUV_{max}	0.92	1.08	**1.00**	1.06	0.83	**0.95**
	TBR_{mean}	1.28	1.11	**1.20**	1.20	1.07	**1.14**
	TBR_{max}	1.58	1.42	**1.50**	1.40	1.18	**1.29**
Tumor VOI size (mm^3	T2-weighted	11.2	13.2	**12.3**	9.8	10.9	**10.4**
	PET	20.2	16.7	**18.5**	5.8	10.7	**8.3**

Tab. S5 Static and dynamic parameters derived from [^{18}F]**AG-120**-PET acquisition

[^{18}F]**AG-120**	parameter	R1-IDH1-WT	R2-IDH1-WT	**Mean IDH1-WT**	R3-IDH1-R132H	R4-IDH1-R132H	**Mean IDH1-R132H**
dynamic parameter (SUV_{mean} TACs)	Time-to-peak (min)	0.83	0.67	**0.75**	1.33	1.00	**1.17**
	TAC peak value (SUV)	0.48	0.43	**0.46**	0.42	0.40	**0.41**
	AUC_{60min} (SUV*min)	8.14	9.17	**8.65**	9.64	10.28	**9.96**
	r^2	0.56	0.77	**-**	0.66	0.77	**-**
	$slope_{30\text{-}60\ min}$ (SUV/min)	-0.0006	-0.0011	**-**	-0.0039	0.0011	**-**
Static parameter (30-60 min)	SUV_{peak}	0.14	0.21	**0.18**	0.17	0.15	**0.16**
	SUV_{max}	0.16	0.27	**0.21**	0.20	0.22	**0.21**
	TBR_{mean}	1.19	1.07	**1.13**	1.33	1.74	**1.54**
	TBR_{max}	1.61	2.17	**1.89**	1.76	2.28	**2.02**
Tumor VOI size (mm^3)	T2-weighted	27.3	12.3	**19.9**	17.0	13.4	**15.2**
	PET	4.4	2.6	**3.5**	24.0	16.9	**20.5**
	T1-weighted + prohance	-	-	**-**	17.1	-	**-**

[1] Popovici-Muller J, Lemieux RM, Artin E, et al. Discovery of AG-120 (Ivosidenib): A First-in-Class Mutant IDH1 Inhibitor for the Treatment of IDH1 Mutant Cancers. ACS Med Chem Lett. 2018;9:300–305. https://doi.org/10.1021/acsmedchemlett.7b00421.

[2] Wang M, Glick-Wilson BE, Zheng Q-H. Facile fully automated radiosynthesis and quality control of O-(2-[18F]fluoroethyl)- -tyrosine ([18F]FET) for human brain tumor imaging. Applied Radiation and Isotopes. 2019;154:108852. https://doi.org/10.1016/j.apradiso.2019.108852.

[3] Bourdier T, Greguric I, Roselt P, Jackson T, Faragalla J, Katsifis A. Fully automated one-pot radiosynthesis of O-(2-[18F]fluoroethyl)-l-tyrosine on the TracerLab FXFN module. Nuclear Medicine and Biology. 2011;38:645–651. https://doi.org/10.1016/j.nucmedbio.2011.01.001.

[4] Antuganov D, Zykov M, Timofeev V, et al. Copper-Mediated Radiofluorination of Aryl Pinacolboronate Esters: A Straightforward Protocol by Using Pyridinium Sulfonates. Eur J Org Chem. 2019;2019:918–922. https://doi.org/10.1002/ejoc.201801514.

[5] Zhang X, Basuli F, Swenson RE. An azeotropic drying-free approach for copper-mediated radiofluorination without addition of base. J Label Compd Radiopharm. 2019;62:139–145. https://doi.org/10.1002/jlcr.3705.

[6] Mossine AV, Brooks AF, Ichiishi N, Makaravage KJ, Sanford MS, Scott PJH. Development of Customized [18F]Fluoride Elution Techniques for the Enhancement of Copper-Mediated Late-Stage Radiofluorination. Sci Rep. 2017;7:233. https://doi.org/10.1038/s41598-017-00110-1.

[7] Makaravage KJ, Brooks AF, Mossine AV, Sanford MS, Scott PJH. Copper-Mediated Radiofluorination of Arylstannanes with [^{18} F]KF. Org Lett. 2016;18:5440–5443. https://doi.org/10.1021/acs.orglett.6b02911.

[8] Tago T, Toyohara J, Ishii K. Preclinical Evaluation of an ^{18} F-Labeled SW-100 Derivative for PET Imaging of Histone Deacetylase 6 in the Brain. ACS Chem Neurosci. 2021;12:746–755. https://doi.org/10.1021/acschemneuro.0c00774.

[9] Zarrad F, Zlatopolskiy B, Krapf P, Zischler J, Neumaier B. A Practical Method for the Preparation of 18F-Labeled Aromatic Amino Acids from Nucleophilic [18F]Fluoride and Stannyl Precursors for Electrophilic Radiohalogenation. Molecules. 2017;22:2231. https://doi.org/10.3390/molecules22122231.

[10] Kessler J, Güttler A, Wichmann H, et al. IDH1(R132H) mutation causes a less aggressive phenotype and radiosensitizes human malignant glioma cells independent of the oxygenation status. Radiother Oncol. 2015;116:381–387. https://doi.org/10.1016/j.radonc.2015.08.007.

[11] Schindelin J, Arganda-Carreras I, Frise E, et al. Fiji: an open-source platform for biological-image analysis. Nat Methods. 2012;9:676–682. https://doi.org/10.1038/nmeth.2019.

[12] Wenzel B, Liu J, Dukic-Stefanovic S, et al. Targeting cyclic nucleotide phosphodiesterase 5 (PDE5) in brain: Toward the development of a PET radioligand labeled with fluorine-18. Bioorganic Chemistry. 2019;86:346–362. https://doi.org/10.1016/j.bioorg.2019.01.037.
[13] Lindemann M, Hinz S, Deuther-Conrad W, et al. Radiosynthesis and in vivo evaluation of a fluorine-18 labeled pyrazine based radioligand for PET imaging of the adenosine A2B receptor. Bioorganic & Medicinal Chemistry. 2018;26:4650–4663. https://doi.org/10.1016/j.bmc.2018.07.045.
[14] Juratli TA, Prilop I, Saalfeld FC, et al. Sporadic multiple meningiomas harbor distinct driver mutations. Acta Neuropathol Commun. 2021;9:8. https://doi.org/10.1186/s40478-020-01113-2.
